# Structure reveals a regulation mechanism of plant outward-rectifying K^+^ channel GORK by structural rearrangements in the CNBD–Ankyrin bridge

**DOI:** 10.1073/pnas.2500070122

**Published:** 2025-07-23

**Authors:** Taro Yamanashi, Yuki Muraoka, Tadaomi Furuta, Tsukasa Kume, Natsuko Sekido, Shunya Saito, Shota Terashima, Takeshi Yokoyama, Yoshikazu Tanaka, Atsushi Miyamoto, Kanane Sato, Tomoyuki Ito, Hikaru Nakazawa, Mitsuo Umetsu, Ellen Tanudjaja, Masaru Tsujii, Ingo Dreyer, Julian I. Schroeder, Yasuhiro Ishimaru, Nobuyuki Uozumi

**Affiliations:** ^a^Department of Biomolecular Engineering, Graduate School of Engineering, Tohoku University, Sendai 980-8579, Japan; ^b^School of Life Science and Technology, Institute of Science Tokyo, Yokohama 226-8501, Japan; ^c^Department of Molecular and Chemical Life Sciences, Graduate School of Life Sciences, Tohoku University, Sendai 980-8577, Japan; ^d^Center of Bioinformatics, Simulation and Modeling, Department of Bioinformatics, Facultad de Ingeniería, Universidad de Talca, Talca 3460000, Chile; ^e^Cell and Developmental Biology Department, School of Biological Sciences, University of California San Diego, La Jolla, CA 92093-0116

**Keywords:** potassium channel, ankyrin, cryo-EM, CNBD

## Abstract

Guard cells, which regulate stomatal apertures in plants, possess a sophisticated mechanism for regulating turgor pressure. The outward-rectifying “K^+^_out_” channel GORK, expressed in guard cells of the plant *Arabidopsis thaliana*, is a central component that promotes stomatal closure by releasing K^+^ to the extracellular space, thereby lowering turgor pressure. To date, the structural basis underlying the regulation of the K^+^ transport activity of GORK is unclear. Using cryo-EM, we determined the structures of the GORK outward-rectifying K^+^ channel with a resolution of 3.16 to 3.27 Å in five distinct conformations that differ significantly in their C-terminal cyclic nucleotide binding domain (CNBD) and ankyrin repeat (ANK) domain. The C-linker connects the transmembrane domains to the C-terminal domains, i.e., CNBD, CNBD–Ankyrin bridge, and ANK. The structural changes and interactions in the C-linker determine whether the closed state of GORK is closer to the preopen state or in a more removed state from the open state of the channel. In particular, interconversion in the short sequence within the CNBD–Ankyrin bridge plays a decisive role in this determination. This region forms an α-helix in the preopened state, while it adopts a nonhelical structure in further distant closed states. The dynamics of the cytosolic region strongly suggest that the K^+^ channel activity of GORK is regulated by cytosolic signaling factors during stomatal closure.

## Significance

The potassium ion efflux channel GORK plays a critical role in closing plant leaf stomata by releasing K^+^ from guard cells to reduce guard cell volume and turgor. Despite its importance, the regulatory mechanisms controlling GORK activity remain unclear. Here, we have resolved distinct three-dimensional cryo-EM structures of GORK and captured its transition from a tightly closed state to a preopened state. Notably, we show that eight amino acids between the cyclic nucleotide domain and ankyrin domain in the cytosolic C-terminus play a key role in distinguishing the preopened state from other closed states. These findings reveal how structural fluctuations in GORK influence its activity, providing insights into the regulatory mechanisms of GORK-mediated K^+^ release critical for stomatal function.

Plants possess sophisticated mechanisms to control stomatal aperture in response to environmental conditions (1, 2). Stomatal aperture is regulated by changes in turgor pressure of a pair of guard cells surrounding the stomata (3, 4). Opening and closing of stomatal pores in leaves and the turgor changes in guard cells require potassium ion (K^+^) accumulation and K^+^ release, respectively (5). Voltage-dependent inward-rectifying K^+^ (K^+^_in_) channels and outward-rectifying K^+^ (K^+^_out_) channels, that both do not rapidly inactivate, can mediate long-term physiological rates of K^+^ uptake and K^+^ release in guard cells during stomatal movements (6–8). Previous studies have shown that the guard cell K^+^_out_ channel senses and shifts its activation voltage in response to K^+^ concentration changes, thus favoring steady-state K^+^ efflux from guard cells (9, 10). K^+^_out_ channels show a higher activity at alkaline cytosolic pH (11, 12). Furthermore, reduction in K^+^_out_ channel activity (“wash out”) during whole-cell patch clamp recording in guard cells indicates important incompletely understood intracellular regulatory mechanisms of this K^+^ channel.

**Fig. 1.**
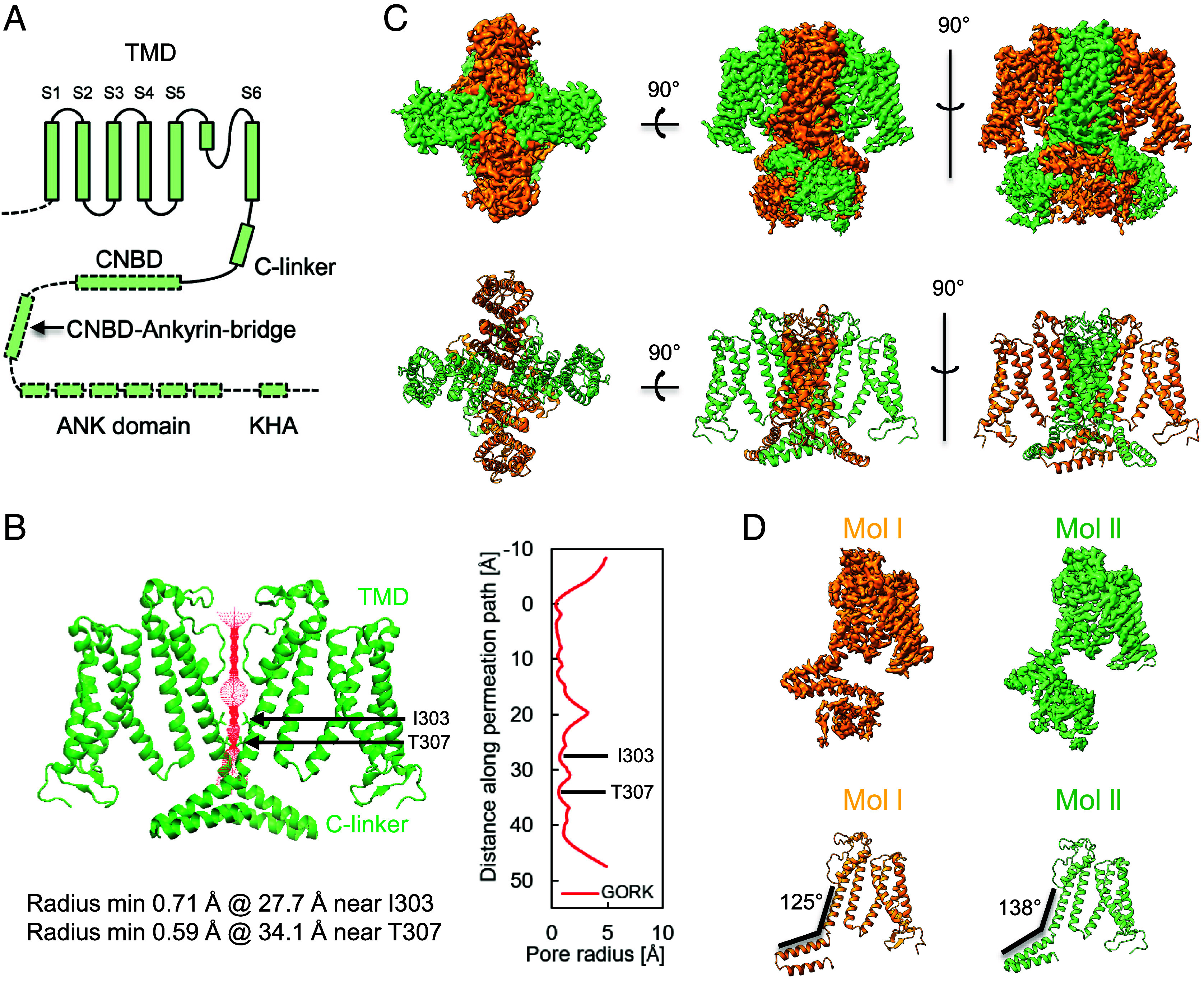
Consensus map of GORK shows rigid transmembrane and flexible cytosolic domain. (*A*) Diagram of the major domains in a GORK subunit. (*B*) Radius of the pore of GORK calculated by the HOLE program. The amino acids restricting the inner gate are shown in sticks (I303 and T307). (*C*) Rigid consensus map of GORK and cartoon representation of a tetramer of Arabidopsis GORK. (*D*) Two different conformations of protomers are named as Mol I/I′ and Mol II/II′ based on their structures. All subunits uniformly indicate depolarized structures.

The GUARD CELL OUTWARD-RECTIFYING K^+^ (GORK) channel is expressed in Arabidopsis guard cells and encodes a K^+^_out_ channel (8, 13, 14). GORK is one of the nine members of the voltage-dependent K^+^ channel family in Arabidopsis (15–19). Of these, the structures of the inward-rectifying K^+^ channels KAT1, AKT1, and the Stelar K^+^ outward rectifier (SKOR) (20) channel have been reported (21–26). Voltage-dependent K^+^ channels, including GORK, contain three regions: an intracellular N-terminal region, a transmembrane region, and an intracellular C-terminal region (27–30). The transmembrane region is composed of six transmembrane helices (S1-S6), with S1-S4 forming the voltage sensing domain and S5 and S6 forming the pore region. Notably, the S4 α-helix is known to be crucial for sensing the electrical membrane voltage (31–33). The C-terminal region is further divided into subdomains: a) a so-called C-linker, b) a cyclic nucleotide binding domain (CNBD), c) the connection domain between CNBD and ankyrin repeat domain (ANK), termed as the CNBD–Ankyrin bridge, d) an ANK, and e) an acidic domain (KHA) (Fig. 1*A*). The *KAT1* gene encodes an inward-rectifying K^+^ channel and does not have an ANK repeat (30, 34). While the C-linker and the CNBD have been reported to play important roles in regulating the gating properties of the inward-rectifying K^+^_in_ channels KAT1 and AKT1 (21–23), the functional significance of the roles of the C-linker/CNBD domains for outward-rectifying K^+^_out_ channels and the downstream CNBD–Ankyrin bridge/ANK/KHA domain remains unclear. In this study, we report the cryo-EM structure of GORK in five different conformations and propose a mechanism that regulates its activity via the CNBD–Ankyrin bridge.

## Results

### Structure of GORK With a Robust Transmembrane Domain and Dynamic Cytoplasmic Region.

The GORK outward-rectifying K^+^ channel protein (UniProt: Q94A76) with Flag tag at the N terminus and His-tag at the C-terminus was produced in baculovirus-infected Sf9 cells and purified to homogeneity with anti-Flag affinity gel and size exclusion chromatography for cryoelectron microscopy (cryo-EM) analysis. A consensus map of GORK at a resolution of 2.55 Å was obtained from 186,257 particles (*SI Appendix*, Fig. S1 and Table S1). The tertiary structure of GORK obtained displayed a closed state. The core of GORK forms a voltage-dependent (*Shaker*-type) channel with a K^+^ conducting pore (Fig. 1*A*), whereby the narrowest part of the conducting pore is located at positions Ile 303 and Thr 307 (Fig. 1*B*). Although the transmembrane domain exhibited a rigid fourfold symmetry (C4 symmetry) map, the electron microscopy (EM) densities of the cytosolic region were faint (23, 25). Consequently, the model structure was built only up to the B′ helices in the C-linker, as described in a previous report on AKT1 (23) (Figs. 1 *C* and *D* and *SI Appendix*, Fig. S2). Based on the cytosolic EM densities, twofold symmetry (C2 symmetry) were identified as diagonal subunits, sharing similar conformations and designated as Mol I/I′ and Mol II/II′. Notably, Mol I and Mol II displayed distinct trends in visibility, suggesting that the conformational differences between Mol I/I′ and Mol II/II′ may result from differing structural regulatory mechanisms (Fig. 1*D*). A prior study elucidated the AKT1 structure up to the CNBD domain, excluding the ANK and KHA domains (21–23). Also in our case, the density map of GORK after the B′ helices in C-linker were insufficient to construct a complete structural model of GORK. Nonetheless, the cytosolic region of GORK was observable at a low contour level (threshold 0.06) (*SI Appendix*, Fig. S1*G*).

**Fig. 2.**
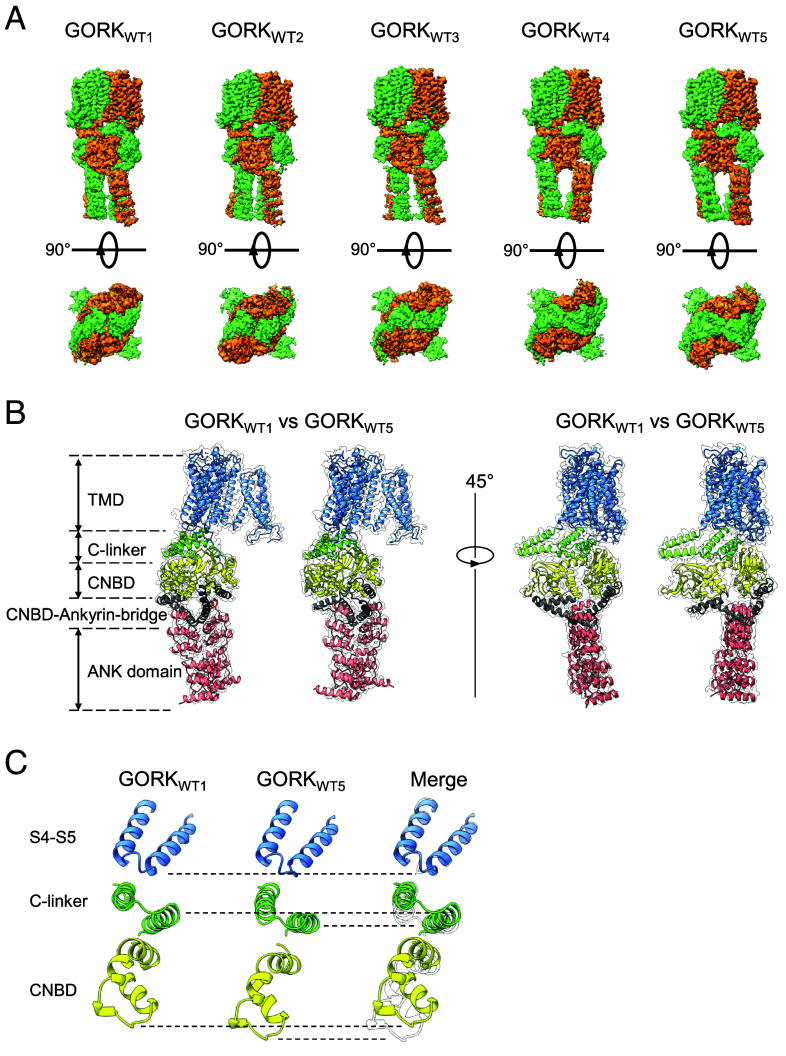
Five different cytosolic conformations of GORK. (*A*) The five different Cryo-EM density maps of almost twofold symmetry viewed from side and the cytoplasmic side. (*B*) Representative comparison of conformational changes in ANK, CNBD–Ankyrin bridge and CNBD regions. (*C*) GORK_WT1_ (*Left*), GORK_WT5_ (*Center*), and their superimposed model (*Right*) of S4 and S5, C-linker domain, and CNBD.

These findings suggested that particles with multiple conformations may have been incorporated into a single structure. For further investigation, a mask for the ANK domain was prepared, and an ANK-focused 3D classification was conducted (*SI Appendix*, Fig. S3). As a result, five different structures of GORK ranging from the transmembrane region to the C-terminal ANK (designated as GORK_WT1_–GORK_WT5_) were determined at resolutions of 3.16 to 3.27 Å (Fig. 2*A* and *SI Appendix*, Fig. S3 and Movie S1). The five GORK structures showed N-terminally an almost uniform transmembrane domain (positions 50 to 313 from the initial Met), including the voltage-sensing domain and the ion selectivity filter. In contrast, the following cytosolic regions, i.e., C-linker (positions 314 to 364), CNBD (positions 365 to 490), CNBD–Ankyrin bridge (positions 491 to 542), and ANK (positions 543 to 722) show five distinct structures in almost twofold symmetry (C2 symmetry) (23, 25). The N-terminal cytosolic region upstream of S1 and the C-terminal cytosolic region downstream of the ANK domain, which contains the acidic KHA domain, could not be resolved probably due to their high flexibility. Interestingly, GORK consists of two pairs of dimers, with each dimer exhibiting a different conformation (*SI Appendix*, Fig. S4). In particular, CNBD, CNBD–Ankyrin bridge, and ANK showed distinct conformations (Fig. 2*B*). Furthermore, overlaying GORK_WT1_ and GORK_WT5_, which had high resolutions of 3.17 and 3.16 Å, respectively, revealed that one of the C-linkers in each dimer was pushed upward by the CNBD in GORK_WT1_, as compared to GORK_WT5_ (Fig. 2*C*).

### Role of the Cytosolic Regions for the C2 Symmetry and C4-like C2 Symmetry Structure.

**Fig. 3.**
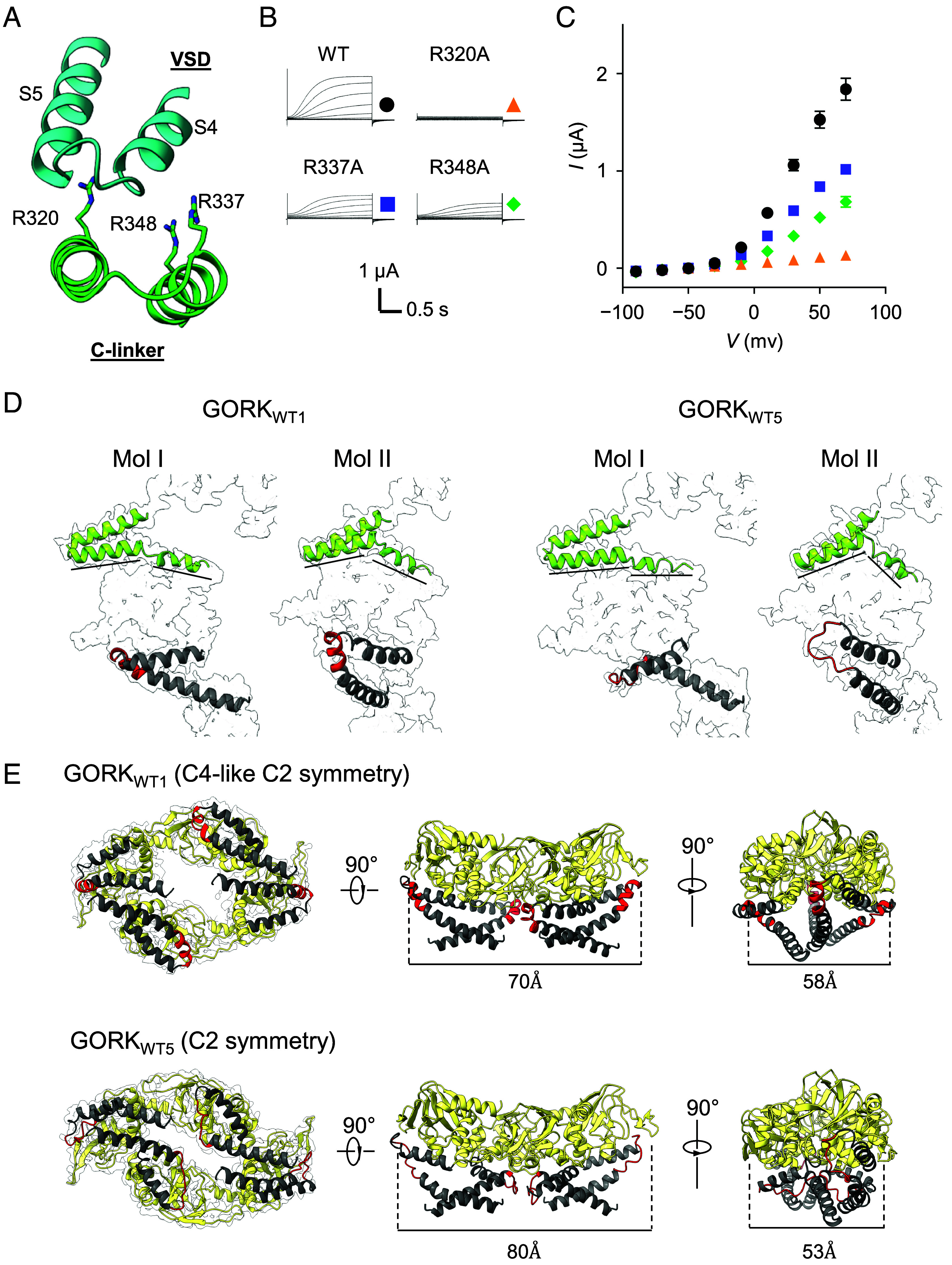
Conformational shift from C2 symmetry to C4-like C2 symmetry structure of GORK. (*A*) The C-linker exposes a few positively charged residues toward the S4 and S5 loop belonging to the voltage sensing domain (VSD). (*B*) Representative current traces of GORK variants. Images have been cropped to leave only the important parts. (*C*) Current–voltage relationship of steady-state currents obtained from *Xenopus* oocytes expressing the indicated GORK variants. Data are mean ± SEM, *n* = 6. (*D*) The important gap needed for the C2 symmetry-C4 symmetry conformational shift after the B′ helices in the C-linker in GORK_WT1_ and GORK_WT5_. (*E*) C4-like C2 symmetry and C2 symmetry of the CNBD in GORK_WT1_ and GORK_WT5_ and drastic structural difference within the CNBD–Ankyrin bridge (residues 511 to 519, red).

Previous structural analyses revealed that the function of KAT1 is affected by interactions between its voltage-sensing domain and its C-linker via arginine (Arg) residues that interact with the S4 and S5 loop (22). GORK has three Arg residues in the C-linker (R320, R337, R348) that face the S4 and S5 loop (Fig. 3*A*). Individual substitution of these residues affected K^+^ transport activity of GORK (Fig. 3 *B* and *C*). The R320A variant completely abolished K^+^ transport ability, while the R337A and R348A variants displayed reduced activity, suggesting that R320/R337/R348 in the C-linker are important for controlling K^+^ transport activity via interaction with the voltage sensing domain. Closer inspection of the structure revealed that the difference of the degree of bending in the C-linker between Mol I and Mol II was small in GORK_WT1_ but greater in GORK_WT5_ (Fig. 3*D*). Additionally, GORK_WT1_ displayed a C4-like C2 symmetry in the CNBD, whereas GORK_WT5_ showed a pronounced C2 symmetry (Fig. 3*E*). Furthermore, we found a structural difference in the short structure that lies between the CNBD and ANK domains, termed as CNBD–Ankyrin bridge containing residues 511 to 519 (Fig. 1*A*). This region had an α-helical structure in GORK_WT1_, while in GORK_WT5_, it was entirely present in a nonhelical loop conformation (Fig. 3 *D* and *E* and Movies S2–S5). The intermediate structures GORK_WT2_, GORK_WT3_, and GORK_WT4_ were mixtures of both extreme structures GORK_WT1_ and GORK_WT5_ (*SI Appendix*, Fig. S5). Thus, the rigidity provided by the α-helix in the CNBD–Ankyrin bridge apparently contributed to the stability toward a C4 symmetry in the C-linker and the CNBD.

### Swap of the Region Downstream the CNBD Between GORK and AKT1.

**Fig. 4.**
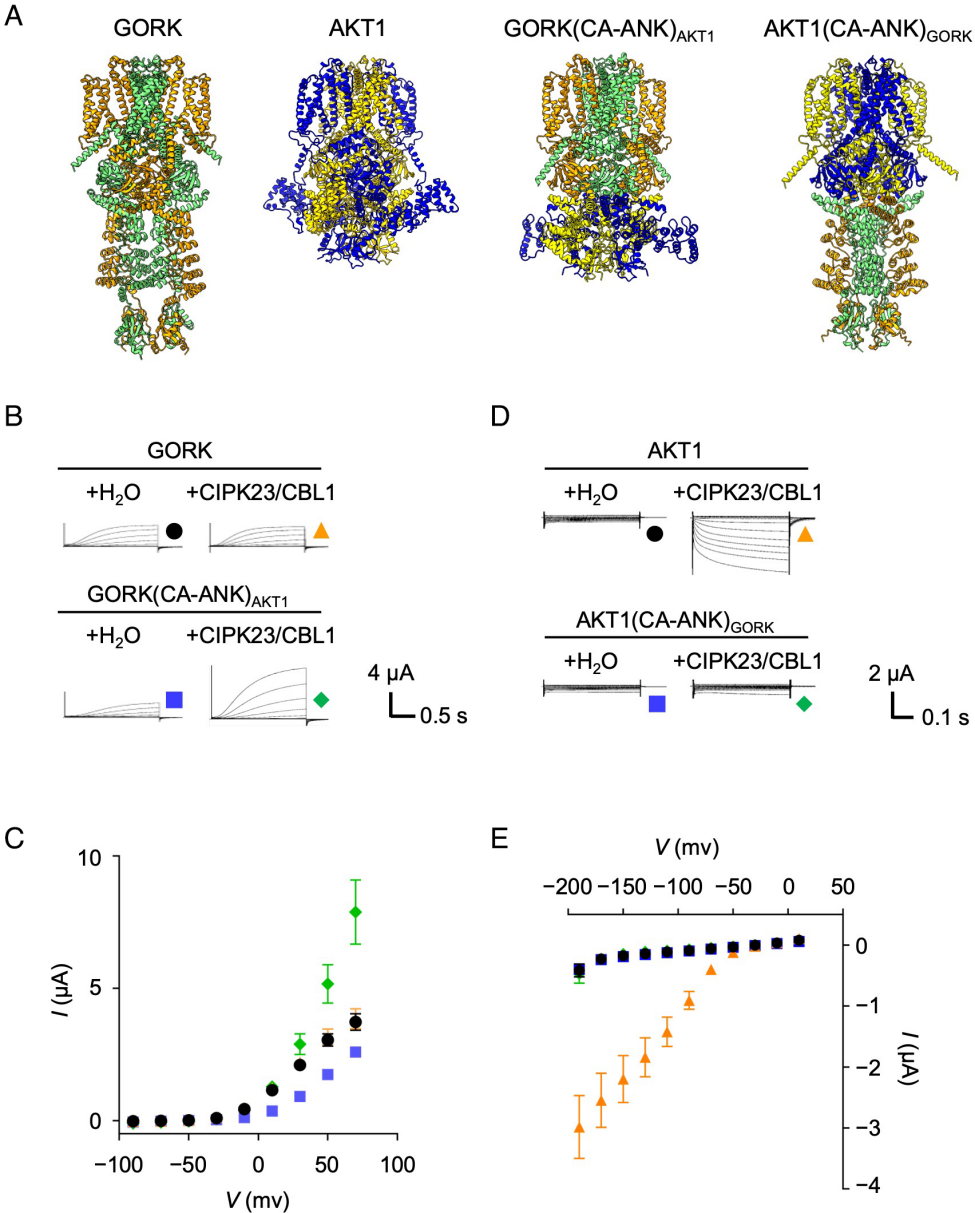
Features of GORK-AKT1 chimeras. (*A*) Hypothetical models of GORK, phosphorylated AKT1, GORK(CA-ANK)_AKT1_, and AKT1(CA-ANK)_GORK_ predicted by Alphafold3. (*B*–*E*) Electrophysiological recordings of GORK-AKT1 chimeras expressed in Xenopus oocytes. (*B* and *D*) Representative current traces of indicated GORK/AKT1 variants. Images have been cropped to leave only the important parts. (*C* and *E*) Current–voltage relationship of steady-state currents versus applied voltages in (*B*) and (*D*). Data are mean ± SEM, *n* = 6.

The inward-rectifying K^+^ channel AKT1 changes its cytosolic structure from C2 symmetry to C4 symmetry prior to final channel opening, especially when it interacts with the CIPK23/CBL1 protein kinase complex (23). AKT1 has an α-helix in the CNBD–Ankyrin bridge in both C2 symmetry and C4 symmetry (*SI Appendix*, Fig. S6 *A* and *B*). GORK_WT1_ displayed a C4-like C2 symmetry with an α-helix in the CNBD–Ankyrin bridge (Fig. 3*E*). From these structural analyses, we hypothesized that transplanting the region downstream of the CNBD (containing CNBD–Ankyrin bridge and ANK) from AKT1 to GORK could create a rigid CNBD–Ankyrin bridge in GORK and confer C4 symmetry to the C-linker and the CNBD when coexpressed with CIPK23/CBL1. Therefore, we constructed the chimeric channel GORK(CA-ANK)_AKT1_, where the CNBD–Ankyrin bridge/ANK/KHA region of GORK was replaced by that of AKT1, as well as the inverse chimera AKT1(CA-ANK)_GORK_ (Fig. 4*A*). GORK(CA-ANK)_AKT1_ exhibited CIPK23/CBL1-dependent stimulation of activation of the outward K^+^ current (roughly threefold increase at 70 mV) (Fig. 4 *B* and *C*). In contrast, AKT1(CA-ANK)_GORK_ was a chimera that almost completely lost its kinase-dependent K^+^ transport activity (Fig. 4 *B*–*E*). Thus, GORK(CA-ANK)_AKT1_ functionally interacted with the CIPK23/CBL1 kinase complex. The higher channel activity may indicate that the kinase influenced the structure in regions in the cytosolic C-terminus promoting C4 symmetry, which appears to be a prerequisite for final voltage-gated channel opening.

### Possible Conformational Change of CNBD–Ankyrin Bridge.

**Fig. 5.**
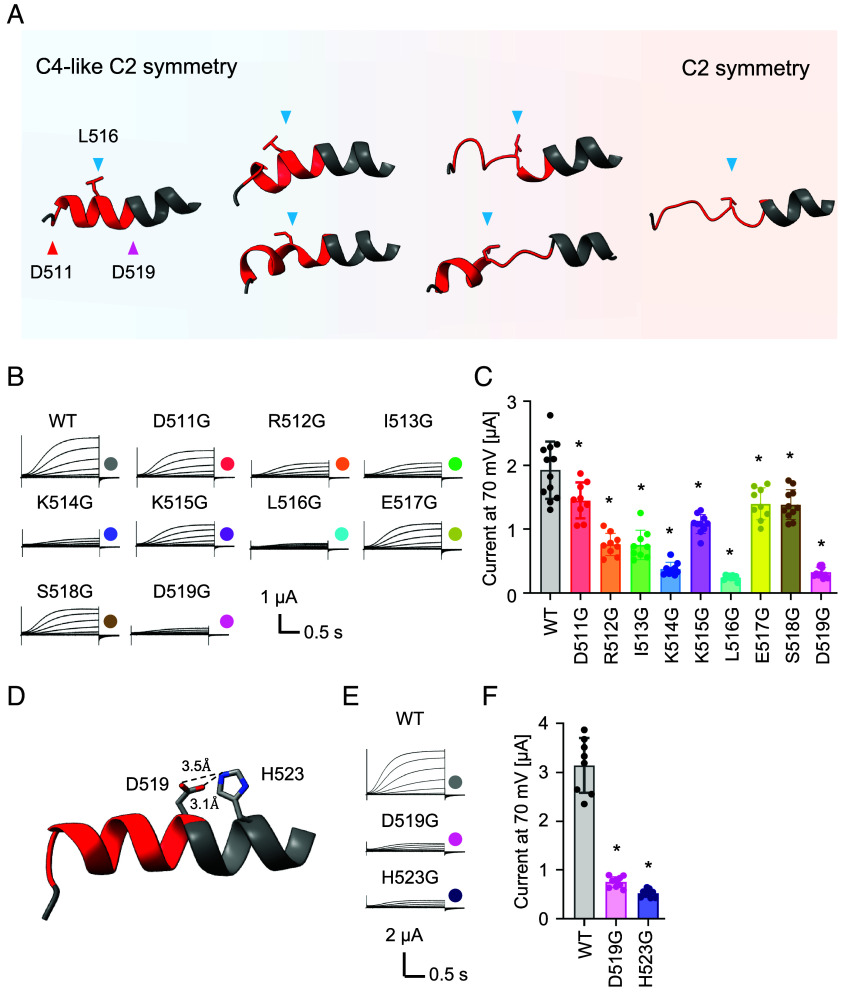
Glycine scanning analysis of the core sequence of the CNBD–Ankyrin bridge. (*A*) Different secondary structures of the CNBD–Ankyrin bridge of five different GORK structures (GORK_WT1_–GORK_WT5_). Intermediate conformation in GORK_WT2_ (*Top-Left*), GORK_WT4_ (*Top-Right*), GORK_WT2_ (*Bottom-Left*), and GORK_WT3_ (*Bottom-Right*). The region of D511–D519 is indicated as red. (*B*) Electrophysiological recordings of GORK variants containing Gly at residues 511to 519 in *Xenopus* oocytes. Representative current traces of indicated GORK variants. Images have been cropped to leave only the important parts. (*C*) Current amplitudes at the end of the +70 mV pulse. Statistical significance was determined by Student's *t* test compared to wild-type (*P < 0.05). (*D*) Hydrogen bond between D519 and H523 in GORK_WT1_. (*E*) Electrophysiological recordings of variants of D519 and H523 in *Xenopus* oocytes. Representative current traces of the indicated GORK variants. Images have been cropped to leave only the important parts. (*F*) Current amplitudes at the end of the +70 mV pulse. Statistical significance was determined by Student's *t* test compared to wild-type (*P < 0.05).

The CNBD–Ankyrin bridges of GORK_WT1_–GORK_WT5_ contain a mixture of α-helical and nonhelical structures, and these conformational changes appear to be centered around L516 (Fig. 5*A*). To gain insight into the structure of the CNBD–Ankyrin bridge, we generated GORK variants with replacements of each residue at positions 511 to 519 with Gly as an α-helix breaker (35). All variants showed a decrease in K^+^ transport activity, but in particular K514G, L516G, and D519G displayed marked decrease in K^+^ current amplitude (Fig. 5 *B* and *C*). The Cryo-EM structural data showed that K514 likely formed salt bridges with E508, E517, and D511 in GORK_WT1_, and that L516 engaged in hydrophobic interaction with I457, P466, and F467 in GORK_WT1_ (*SI Appendix*, Fig. S9). Additionally, D519 formed hydrogen bonds with H523 within the same α-helical structure (Fig. 5*D*). To corroborate this possibility, we examined the K^+^ channel activity of the H523G variant. The K^+^ channel activity of H523G decreased to a level similar to D519G (Fig. 5 *E* and *F*). D519 and H523 likely contributed to enhancing the α-helix stability of the CNBD–Ankyrin bridge. These data suggest that alterations in the amino acid interactions involving K514, L516, and D519 affect channel activity by interfering with the conformational changes in the secondary structure of the CNBD–Ankyrin bridge.

## Discussion

Based on five different resolved outward-rectifying K^+^ channel GORK structures, this study reveals the fourfold symmetry (C4 symmetry) structure of the transmembrane domain of GORK, and intermediates between C4-like twofold symmetry (C4-like C2 symmetry) and twofold symmetry (C2 symmetry) structures of the C-terminal cytosolic regions (23, 25) (Figs. 1–4). The rigidity of the CNBD–Ankyrin bridge appeared to correlate with the structure of the C-linker and CNBD, determining a C2 symmetry or C4 symmetry of the cytosolic region in GORK. The alteration between α-helical and nonhelical structure of the CNBD–Ankyrin bridge may be affected by the interaction of K514, L516, and D519 with other amino acids (Fig. 5 and *SI Appendix*, Fig. S9). Upon coexpression with CIPK23/CBL1, the cryo-EM-derived AKT1 structure reveals phosphorylation in the N terminus and C-linker of AKT1, as well as additional phosphorylation in the C-terminal region by other kinases that cannot be definitively attributed to CIPK23/CBL1 (23, 36). These residues are not conserved in GORK, indicating that phosphorylation may not be the main factor for channel upregulation. However, we cannot exclude that other residues in the GORK(CA-ANK)_AKT1_ chimera might be targets of CIPK23/CBL1 or endogenous kinases in *Xenopus laevis* oocytes. CPK21 phosphorylates S518 of the CNBD–Ankyrin bridge in GORK in response to a cytosolic Ca^2+^ rise in guard cells induced by ABA under drought conditions (37) (Fig. 5). The lysine and the phosphoserine (pS) would stabilize the α-helical structure through charge-reinforced side chain interactions (38). Molecular dynamics simulation indicated that pS518 forms a salt bridge with K515, but not with K514 (*SI Appendix*, Fig. S10 *A* and *B*). We also examined the secondary structure of the phosphorylated CNBD–Ankyrin bridge peptides, termed as pS518 peptide, and K515G-containing pS518 peptide, termed as K515G-pS518 peptide, by circular dichroism spectroscopy. The α-helix content of the K515G-pS518 peptide was lower than that of the pS518 peptide (*SI Appendix*, Fig. S10 *C* and *D*). The salt bridge between K515 and pS518 might stabilize the α-helix structure of CNBD–Ankyrin bridge. The glycine scanning approach also implied a contribution of D519 to enhancing the α-helix stability of the CNBD–Ankyrin bridge (Fig. 5). Based on the cryo-EM results, the region around K514 and L516 shows changes in secondary structure, while the region around D519 maintains its helical structure (Fig. 5*A*). This result likely accounts for why, even if the peptide around K514 and L516 changes to α-helical or nonhelical structures, the sequence from D519 onward retains its propensity to form a stable α-helical structure. In addition to phosphorylation, the kinase complex (including an inactive kinase variant) is proposed to exert an effect on the structure of the CNBD–Ankyrin bridge of AKT1 that coincides with the transition of the channel to a preopen state with a C4 symmetry of the C-terminal cytosolic domains (23, 36). Our structural and functional data correlate with and specify this notion for GORK. The present findings indicate that the rigidity of the CNBD–Ankyrin bridge may regulate K^+^ channel activity of GORK by controlling the C4 symmetry and C2 symmetry of the C-linker and the CNBD.

The CNBD–Ankyrin bridge is connected to the ANK domain, and strikingly, the ANK domains of the five GORK structures showed the most significant structural differences (Fig. 2 *A* and *B* and 3*D* and *SI Appendix*, Figs. S3–S5). Therefore, there may be a functional causality between ANK and structural changes in the CNBD–Ankyrin bridge. ANK domains are among the most common structural motifs in proteins and mediate protein–protein interactions (39). Nevertheless, ion channels containing ANK domains are limited in number, found only in animal temperature-sensitive TRP and plant voltage-dependent K^+^ channels. In TRPV3, a member of the TRP channel family, structural studies suggest that ANK is important for the temperature-dependent activation of transport activity (40). In contrast, ANK is not important for the temperature sensitivity of plant voltage-dependent K^+^ channels of the SKOR-type (41). Two distinct structures have been determined for SKOR in a closed configuration (25), and like GORK, also the SKOR tetramer possesses C2 symmetry. In both SKOR structures that were enlarged by us by modeling, we identify differences in their CNBD–Ankyrin bridge, indicating a certain flexibility of this domain (25) (*SI Appendix*, Fig. S7). In GORK, we can now relate this flexibility to the flexibility in the ANK domain, which could not be resolved in previous studies on plant K^+^ channels.

The ANK domain of plant K^+^ channels has been identified as an important site of interaction with regulatory phosphatases and kinases (42, 43). Our study suggests an additional role in K^+^ channel regulation besides phosphorylation. It is unlikely that the effect of CIPK23/CBL1 on the GORK(CA -ANK)_AKT1_ chimera is due to phosphorylation (Fig. 4). Both channels, GORK and AKT1, have no remarkably conserved common phosphorylation sites. Instead, the transplanted C-terminal region containing the CNBD–Ankyrin bridge and ANK from AKT1 is necessary for the binding of CIPK23 to the chimera (42). The simplest explanation of our data is that this binding alone is likely sufficient to facilitate the conversion of the C-terminal cytosolic region to a preopen state. In such a scenario, the binding of CIPK23/CBL1 to the ANK domain then feeds back to the CNBD–Ankyrin bridge and modifies its rigidity/flexibility, which in turn affects the symmetry of the C-linker and the CNBD. A similar phosphorylation-independent structural role is proposed for the activating effect of the CIPK6/CBL4 complex on the Arabidopsis K^+^ channel AKT2 (44).

The Arabidopsis inward-rectifying K^+^ channels KAT1, KAT2, and AtKC1 and their orthologs from other angiosperms lack an ANK domain. They have lost them during their recent evolutionary specialization (17). Interestingly, the loss of the ANK domain in KAT1, KAT2, and AtKC1 is accompanied with charge neutralizations in those residues that correspond to AKT1-D379 in the C-linker (KAT1-N384, KAT2-N384, AtKC1-G413). All other ANK-containing Arabidopsis K^+^ channels conserved negatively charged aspartate or glutamate residues at that position (GORK-E393, AKT2-E401, AKT5-D403, SPIK-D405, SKOR-E410). The AKT1-D379A was identified as a constitutively active variant, which could mediate K^+^ currents without CIPK23 and CBL1 (23, 36). Thus, KAT1 and KAT2 in particular appear to have lost an intermediate step in the control of channel activity. KAT1 has been reported to adopt a C4 symmetry, with no evidence of it adopting a C2 symmetry (21, 22). These K^+^_in_ channels can respond directly to the membrane voltage, whereas GORK and potentially AKT1and SKOR require a further gating step to be in a preopened state before the voltage can finally take effect.

A study that emerged during our manuscript's review reports four GORK cryo-EM structures at 2.43 to 3.14 Å resolution (45). Our present study reveals five distinct GORK structures and conformational states (at 3.16 to 3.27 Å), all derived from one highly stable structure (at 2.55 Å) and crucially identifies important transition states in the CNBD–Ankyrin bridge domain across different closed channel conformations. Furthermore, our comprehensive electrophysiological analyses of GORK-AKT1 chimeras and systematic Glycine scanning experiments of the CNBD–Ankyrin bridge highlight the critical role of this nine-amino acid bridge domain connecting the CNBD and ANK domains in K^+^ channel regulation, specifically in distinguishing the channel’s preopen state from other conformational states. Collectively, these complementary studies significantly advance our understanding of K^+^_out_ channel regulation during stomatal closure.

In addition to the important flexibility of the cytosolic C-terminus, our structural determination of GORK provides further insights into the core channel structure of outward-rectifying K^+^ channels such as GORK and SKOR, including their K^+^ conducting pore. Remarkably, there are three conserved charged amino acid residues in plant outward K^+^ channels near the pore that are not present in plant inward-rectifying K^+^ channels such as AKT1 or KAT1 (*SI Appendix*, Fig. S8): i) a glutamate residue in the S4 and S5 loop (GORK-E208, SKOR-E225) below the selectivity filter is located in the region that interacts with the C-linker during voltage-gating in both K^+^_in_ and K^+^_out_ channels (21, 22, 25). Nevertheless, K^+^_in_ channels have uncharged residues at that position (e.g., KAT1-T205, AKT1-T198). ii) A lysine residue in a stretch of the S5 and S6 loop (GORK-K240, SKOR-K257) above the selectivity filter that it is not present in KAT1 or AKT1. And iii) an aspartate residue in the S6 helix (GORK-D295, SKOR-D312) next to the selectivity filter has been shown in SKOR to be an essential part of the K^+^-sensor (46) and replacement of the SKOR-D312-M313-I314 motif by the NLG motif found in KAT1 abolished the outward-rectification of the channel (47). This spatial and functional proximity between the permeation pathway and the gate suggests a sophisticated coupling and a highly efficient solution for the K^+^-sensitive gating mechanism of GORK-like K^+^_out_ channels (9, 10). Elucidating the mechanism of ion channel rectification remains an important question for the future.

## Materials and Methods

### Protein Expression and Purification.

The optimized coding DNAs for GORK (Uniprot: Q94A76) from *Arabidopsis thaliana* with the sequence encoding Flag tag at the N terminus and His tag at the C-terminus was synthesized by GenScriptJapan. The *GORK* gene was cloned into a pFastBac-Dual vector. Baculovirus-infected Sf9 cells were used for overexpression and were grown at 27 °C at 120 rpm in the medium Sf-900 III SFM (Thermo Fisher). P2 virus transfected cells were cultured for 48 h before harvesting. Cell pellet from 1 L of culture was resuspended in extraction buffer (10 mM lauryl maltose neopentyl glycol (LMNG), 2 mM cholesteryl hemisuccinate (CHS), 300 mM KCl, 20 mM Tris-HCl pH 8.0, and protease inhibitor cocktail (Roche)) and incubated at 4 °C for 1.5 h. Solubilized membranes were collected by centrifugation at 20,000×*g* for 30 min at 4 °C. The 50 mL of supernatant was applied to 1 mL of anti-Flag M2 affinity gel (Sigma) and rotated for 1 h at 4 °C. The resin was rinsed with the 15 column volume (CV) of wash buffer (0.04% GDN, 300 mM KCl, and 20 mM Tris-HCl pH 8.0) and transferred to the open column after being rinsed with the 10 CV of wash buffer again. The target proteins were eluted with 4 CV of wash buffer supplemented with 200 μg/ml 3 × FLAG peptide. The eluent was concentrated by Amicon Ultra centrifugal filter (MWCO 100 kDa) and then injected to a Superose 6 increase column (GE Healthcare) equilibrated with SEC buffer (0.02% GDN, 150 mM KCl, 20 mM Tris pH 8.0, and 2 mM DTT) followed by centrifugation at 20,000×*g* for 5 min at 4 °C. Peak fractions were pooled and concentrated to 5.9 mg/ml.

### Single-Particle cryo-EM Data Acquisition.

Purified protein (3 μl) at a concentration of 4 mg/ml was added to the freshly plasma-cleaned holey carbon grids (Quantifoil, R1.2/1.3, 200 mesh, Cu). The grids were blotted for 3 s at 100% humidity and 4 °C with the use of Vitrobot Mark IV (ThermoFisher Scientific) and plunge-frozen into liquid ethane cooled by liquid nitrogen. The blotted grids were stored in liquid nitrogen until imaging. Grids were transferred to CRYO ARM 300 II (JEOL) operated at 300 kV. Movie micrographs were collected at 60,000 x nominal magnification (corresponding to a physical pixel size of 0.788 Å) with a total dose of 60 electrons per Å^2^ using a K3 Camera direct electron detector (Gatan) with the SerialEM program and used for subsequent data processing.

### Image Processing.

In total, 4,675 micrographs were collected using a K3 Camera direct electron detector (Gatan) with the SerialEM program. All data processing for the dataset was performed using CryoSPARC version 4.4. Briefly, Patch Motion Correction and Patch CTF Estimation were performed, and then Manually Curate Exposures was used to eliminate disqualified micrographs determined by thresholding and manual selection. Particle picking was performed on the selected micrographs using Template Picker, extracted with a box size of 320 × 320 pixels (*SI Appendix*, Fig. S1*A*). The extracted particles were applied to two-dimensional (2D) classifications, and most of 2D classes indicated a similar 2D model of another outward channel, SKOR (*SI Appendix*, Fig. S1*B*). After all particles are sorted again by Extract from Micrographs, in total, 186,257 particles were used for Nonuniform Refinement, yielding a final reconstruction of GORK consensus map at an overall resolution of 2.55 Å (*SI Appendix*, Fig. S1 *E* and *F* and Table S1). Focused 3D Classification was performed on the ANK domain, and five different conformational maps were obtained. Nonuniform Refinement was performed to obtain five final maps.

### Model Building.

The coordinate of GORK built by Alphafold3 was roughly fitted into the 3D EM maps of GORK using UCSF ChimeraX. Every residue was manually examined. The chemical properties of amino acids were considered during model building. The model was subjected to iterative manual rebuilding using Coot software and real-space refinement with the use of PHENIX. The N-terminal residues before about position 50 and the C-terminal residues after approximately position 720 were truncated due to the lack of corresponding density maps. All the figures in this article were prepared using PyMOL (Schrödinger, LLC.), UCSF Chimera and ChimeraX, VMD (University of Illinois at Urbana-Champaign), and BIOVIA Discovery Studio 2017 R2 (Dassault Systèmes).

### Molecular Dynamics Simulation.

Molecular dynamics (MD) simulation was performed using Amber22 (48) with the Amber ff19SB force field (protein), the TIP3P model (water), and the recent phosphorylated amino acid parameters (pS518) (49). The partial structure of the CNBD–Ankyrin bridge (Gly496-Gly525, 30 residues) as a helix–loop–helix (HLH) was first extracted from wild-type GORK (WT1, PDB 9L9U) obtained in this study. Ser518 was phosphorylated for the pS518 system. The protonation states of ionized residues at pH 7 were determined using the PDB2PQR server, and thus, H523 was protonated (50). The N- and C-termini of the partial structures were capped with Ace and Nme groups, respectively, and then, the system was solvated in a TIP3P water box. For the system, 300 steps of energy minimization with heavy atom positional restraints were performed, followed by equilibration for 500 ps under NVT conditions (300 K) and 500 ps under NPT conditions (300 K and 1 bar) with the same restraints. Finally, a 35 ns production run was performed.

### Electrophysiological Experiments using *Xenopus laevis* Oocytes.

For expression in *Xenopus laevis* oocytes, the cDNAs encoding GORK, AKT1, and their variants were subcloned into pOMU-HESB vector. The GORK/AKT1 mutants in pOMU-HESB were constructed using the In-Fusion HD cloning kit (639648; Takara Bio, Shiga, Japan) in combination with PCR amplification and restriction enzyme analyses as described previously. CBL1 and CIPK23 were subcloned into pGEMKN vectors. cRNAs were synthesized from linearized plasmids using an in vitro transcription kit (mMESSAGE mMACHINE™ T7 Transcription Kit, Ambion, TX). *Xenopus laevis* oocytes were microinjected with 10 ng of cRNA in 50 nL per oocyte and then incubated for 60 to 72 h in Barth’s buffer containing 88 mM NaCl, 1 mM KCl, 0.41 mM CaCl_2_, 0.33 mM Ca(NO_3_)_2_, 1 mM MgSO_4_, 2.4 mM NaHCO_3_, 5 mM Tris-HCl (pH 7.4), and 50 mg/L gentamicin sulfate at 18 °C. During two-electrode voltage clamp recordings for measuring outward K^+^ currents, oocytes were bathed in a solution composed of 12 mM KCl, 108 mM NaCl, 1mM MgCl_2_, 1mM CaCl_2_, and 10 mM HEPES-Tris (pH7.4). Volage clamp protocols applied were as follows: −90 mV to +70 mV, 20 mV increments, 2,000 ms test pulse with holding potential of −100 mV. For measuring inward K^+^ currents, oocytes were bathed in a solution composed of 120 mM KCl, 1mM MgCl_2_, 1mM CaCl_2_, 1mM LaCl_3_, and 10 mM HEPES-NaOH (pH7.3). Voltage clamp protocols applied were as follows: +10 mV to −170 or −190 mV, −20 mV decrements, 500 ms test pulse with holding potential of −40 mV. Recordings and data analysis were performed using an AxoClamp 2B amplifier (Molecular Devices, CA) and an Axon Digidata 1550 System (Molecular Devices).

### Circular Dichroism (CD) Spectroscopy Measurements.

CNBD–Ankyrin bridge peptides (pS518 peptide, EKESNDRIKKLEpSDIVIHIG) and K515G-containing E506-pS518-G525 peptide (K515G-pS518 peptide, EKESNDRIKGLEpSDIVIHIG) were synthesized by GenScript Co. (U.S.A.). The peptides were dissolved in double-distilled water to prepare 10 mM stock solutions. These solutions were then diluted to a final concentration of 50 µM in 1 mM potassium phosphate buffer containing 50 mM KCl (adjusted to pH 6.0 with KOH). CD spectroscopy measurements were carried out with J-820 (JASCO Co., Japan) and secondary structure analysis was performed by online tool BeStSel (https://bestsel.elte.hu/index.php).

## Supplementary Material

Appendix 01 (PDF)

Movie S1. Horizontal view of tetramer.

Movie S2. Vertical view of CNBD-Ankyrin-bridge and CNBD.

Movie S3. Horizontal view of CNBD-Ankyrin-bridge and CNBD.

Movie S4. Horizontal view of tetramer I.

Movie S5. Horizontal view of tetramer II.

## Data Availability

All study data are included in the article and/or supporting information.

## References

[1] T.-H. Kim, M. Böhmer, H. Hu, N. Nishimura, J. I. Schroeder, Guard cell signal transduction network: Advances in understanding abscisic acid, CO_2_, and Ca^2+^ signaling. Ann. Rev. Plant Biol. **61**, 561–591 (2010). 20192751 10.1146/annurev-arplant-042809-112226 PMC3056615

[2] R. Hedrich, D. Geiger, Biology of SLAC1-type anion channels—From nutrient uptake to stomatal closure. New Phytol. **216**, 46–61 (2017). 28722226 10.1111/nph.14685

[3] M. Jezek, M. R. Blatt, The membrane transport system of the guard cell and its integration for stomatal dynamics. Plant Physiol. **174**, 487–519 (2017). 28408539 10.1104/pp.16.01949 PMC5462021

[4] Y. Murata, I. C. Mori, S. Munemasa, Diverse Stomatal Signaling and the Signal Integration Mechanism. Annu. Rev. Plant Biol. **66**, 369–392 (2015). 25665132 10.1146/annurev-arplant-043014-114707

[5] G. D. Humble, K. Raschke, Stomatal opening quantitatively related to potassium transport: Evidence from electron probe analysis 1. Plant Physiol. **48**, 447–453 (1971). 16657817 10.1104/pp.48.4.447 PMC396885

[6] J. I. Schroeder, K. Raschke, E. Neher, Voltage dependence of K+ channels in guard-cell protoplasts. Proc. Natl. Acad. Sci. **84**, 4108–4112 (1987). 16593851 10.1073/pnas.84.12.4108 PMC305032

[7] J. I. Schroeder, H. H. Fang, Inward-rectifying K^+^ channels in guard cells provide a mechanism for low-affinity K^+^ uptake. Proc. Natl. Acad. Sci. **88**, 11583–11587 (1991). 1763075 10.1073/pnas.88.24.11583 PMC53180

[8] E. Hosy , The Arabidopsis outward K^+^ channel GORK is involved in regulation of stomatal movements and plant transpiration. Proc. Natl. Acad. Sci. U.S.A. **100**, 5549–5554 (2003). 12671068 10.1073/pnas.0733970100 PMC154382

[9] J. I. Schroeder, K^+^ transport properties of K^+^ channels in the plasma membrane of Vicia faba guard cells. J. Gen. Physiol. **92**, 667–683 (1988). 3235976 10.1085/jgp.92.5.667 PMC2228917

[10] M. R. Blatt, D. Gradmann, K^+^-sensitive gating of the K+ outward rectifier in *Vicia* guard cells. J. Membr. Biol. **158**, 241–256 (1997). 9263886 10.1007/s002329900261

[11] M. R. Blatt, F. Armstrong, K^+^ channels of stomatal guard cells: Abscisic-acid-evoked control of the outward rectifier mediated by cytoplasmic pH. Planta **191**, 330–341 (1993).

[12] H. Miedema, S. M. Assmann, A membrane-delimited effect of internal pH on the K+ outward rectifier of *Vicia faba* guard cells. J. Membr. Biol. **154**, 227–237 (1996). 8952952 10.1007/s002329900147

[13] P. Ache , GORK, a delayed outward rectifier expressed in guard cells of *Arabidopsis thaliana*, is a K^+^-selective, K^+^-sensing ion channel. FEBS Lett. **486**, 93–98 (2000). 11113445 10.1016/s0014-5793(00)02248-1

[14] G. D. Adem , A master switch of plant metabolism?. Trends Plant Sci. **25**, 434–445 (2020). 31964604 10.1016/j.tplants.2019.12.012

[15] I. Dreyer, N. Uozumi, Potassium channels in plant cells. FEBS J. **278**, 4293–4303 (2011). 21955642 10.1111/j.1742-4658.2011.08371.x

[16] R. Hedrich, Ion channels in plants. Physiol. Rev. **92**, 1777–1811 (2012). 23073631 10.1152/physrev.00038.2011

[17] I. Dreyer , How to grow a tree: Plant voltage-dependent cation channels in the spotlight of evolution. Trends Plant Sci. **26**, 41–52 (2021). 32868178 10.1016/j.tplants.2020.07.011

[18] O. Pantoja, Recent advances in the physiology of ion channels in plants. Annu. Rev. Plant Biol. **72**, 463–495 (2021). 33428476 10.1146/annurev-arplant-081519-035925

[19] P. Maser , Phylogenetic relationships within cation transporter families of Arabidopsis. Plant Physiol. **126**, 1646–1667 (2001). 11500563 10.1104/pp.126.4.1646 PMC117164

[20] F. Gaymard , Identification and disruption of a plant shaker-like outward channel involved in K^+^ release into the xylem sap. Cell **94**, 647–655 (1998). 9741629 10.1016/s0092-8674(00)81606-2

[21] M. D. Clark, G. F. Contreras, R. Shen, E. Perozo, Electromechanical coupling in the hyperpolarization-activated K^+^ channel KAT1. Nature **583**, 145–149 (2020). 32461693 10.1038/s41586-020-2335-4 PMC7747229

[22] S. Li , Cryo-EM structure of the hyperpolarization-activated inwardly rectifying potassium channel KAT1 from Arabidopsis. Cell Res. **30**, 1049–1052 (2020). 32901112 10.1038/s41422-020-00407-3 PMC7784887

[23] Y. Lu , Structural basis for the activity regulation of a potassium channel AKT1 from Arabidopsis. Nat Commun **13**, 5682 (2022). 36167696 10.1038/s41467-022-33420-8 PMC9515098

[24] M. S. Dickinson, S. Pourmal, M. Gupta, M. Bi, R. M. Stroud, Symmetry reduction in a hyperpolarization-activated homotetrameric ion channel. Biochemistry **61**, 2177–2181 (2022). 34964607 10.1021/acs.biochem.1c00654 PMC9931035

[25] S. Li , Cryo-EM structure reveals a symmetry reduction of the plant outward-rectifier potassium channel SKOR. Cell Discov. **9**, 67 (2023). 37391403 10.1038/s41421-023-00572-w PMC10313817

[26] X. Gao , Structural changes in the conversion of an *Arabidopsis* outward-rectifying K+ channel into an inward-rectifying channel. Plant Commun. **5**, 100844 (2024). 38341617 10.1016/j.xplc.2024.100844 PMC11211230

[27] T. Jegla, G. Busey, S. M. Assmann, Evolution and structural characteristics of plant voltage-gated K^+^ channels. Plant Cell **30**, 2898–2909 (2018). 30389753 10.1105/tpc.18.00523 PMC6354262

[28] J. R. Whicher, R. MacKinnon, Structure of the voltage-gated K^+^ channel Eag1 reveals an alternative voltage sensing mechanism. Science **353**, 664–669 (2016). 27516594 10.1126/science.aaf8070 PMC5477842

[29] H. Sentenac , Cloning and expression in yeast of a plant potassium ion transport system. Science **256**, 663–665 (1992). 1585180 10.1126/science.1585180

[30] J. A. Anderson, S. S. Huprikar, L. V. Kochian, W. J. Lucas, R. F. Gaber, Functional expression of a probable Arabidopsis thaliana potassium channel in Saccharomyces cerevisiae. Proc. Natl. Acad. Sci. U.S.A. **89**, 3736–3740 (1992). 1570292 10.1073/pnas.89.9.3736 PMC525565

[31] V. S. Mandala, R. Mackinnon, Voltage-sensor movements in the Eag Kv channel under an applied electric field. Proc. Natl. Acad. Sci. **119**, e2214151119 (2022). 36331999 10.1073/pnas.2214151119 PMC9674223

[32] W. A. Catterall, Ion channel voltage sensors: Structure, function, and pathophysiology. Neuron **67**, 915–928 (2010). 20869590 10.1016/j.neuron.2010.08.021 PMC2950829

[33] X. Tao, A. Lee, W. Limapichat, D. A. Dougherty, R. Mackinnon, A gating charge transfer center in voltage sensors. Science **328**, 67–73 (2010). 20360102 10.1126/science.1185954 PMC2869078

[34] D. P. Schachtman, J. I. Schroeder, W. J. Lucas, J. A. Anderson, R. F. Gaber, Expression of an inward-rectifying potassium channel by the Arabidopsis KAT1 cDNA. Science **258**, 1654–1658 (1992). 8966547 10.1126/science.8966547

[35] L. Serrano, J.-L. Neira, J. Sancho, A. R. Fersht, Effect of alanine versus glycine in α-helices on protein stability. Nature **356**, 453–455 (1992). 1557131 10.1038/356453a0

[36] L. Li, B.-G. Kim, Y. H. Cheong, G. K. Pandey, S. Luan, A Ca^2+^ signaling pathway regulates a K^+^ channel for low-K response in *Arabidopsis*. Proc. Natl. Acad. Sci. **103**, 12625–12630 (2006). 16895985 10.1073/pnas.0605129103 PMC1567929

[37] P. J. M. van Kleeff , The *Arabidopsis* GORK K^+^-channel is phosphorylated by calcium-dependent protein kinase 21 (CPK21), which in turn is activated by 14-3-3 proteins. Plant Physiol. Biochem. **125**, 219–231 (2018). 29475088 10.1016/j.plaphy.2018.02.013

[38] B. M. Baynes, D. I. C. Wang, B. L. Trout, Role of arginine in the stabilization of proteins against aggregation. Biochemistry **44**, 4919–4925 (2005). 15779919 10.1021/bi047528r

[39] L. K. Mosavi, T. J. Cammett, D. C. Desrosiers, Z. Y. Peng, The ankyrin repeat as molecular architecture for protein recognition. Protein Sci. **13**, 1435–1448 (2004). 15152081 10.1110/ps.03554604 PMC2279977

[40] K. D. Nadezhdin , Structural mechanism of heat-induced opening of a temperature-sensitive TRP channel. Nat. Struct. Mol. Biol. **28**, 564–572 (2021). 34239124 10.1038/s41594-021-00615-4 PMC8283911

[41] Y. Muraoka , An outward-rectifying plant K^+^ channel SPORK2 exhibits temperature-sensitive ion-transport activity. Curr. Biol. **33**, 5488–5494 e5487 (2023). 38016479 10.1016/j.cub.2023.10.057

[42] S. C. Lee , A protein phosphorylation/dephosphorylation network regulates a plant potassium channel. Proc. Natl. Acad. Sci. **104**, 15959–15964 (2007). 17898163 10.1073/pnas.0707912104 PMC2000415

[43] I. Cherel , Physical and functional interaction of the Arabidopsis K^+^ channel AKT2 and phosphatase AtPP2CA. Plant Cell **14**, 1133–1146 (2002). 12034902 10.1105/tpc.000943 PMC150612

[44] K. Held , Calcium-dependent modulation and plasma membrane targeting of the AKT2 potassium channel by the CBL4/CIPK6 calcium sensor/protein kinase complex. Cell Res. **21**, 1116–1130 (2011). 21445098 10.1038/cr.2011.50 PMC3193494

[45] X. Zhang , GORK K^+^ channel structure and gating vital to informing stomatal engineering. Nat. Commun. **16**, 1961 (2025). 40000640 10.1038/s41467-025-57287-7 PMC11861651

[46] I. Johansson , External K^+^ modulates the activity of the Arabidopsis potassium channel SKOR via an unusual mechanism. Plant J. **46**, 269–281 (2006). 16623889 10.1111/j.1365-313X.2006.02690.x

[47] F. Poree , Plant K(in) and K(out) channels: Approaching the trait of opposite rectification by analyzing more than 250 KAT1-SKOR chimeras. Biochem. Biophys. Res. Commun. **332**, 465–473 (2005). 15894288 10.1016/j.bbrc.2005.04.150

[48] D. A. Case , Amber 2022 (University of California, San Francisco, 2022).

[49] L. E. Raguette , PHosaa14SB and PHosaa19SB: Updated amber force field parameters for phosphorylated amino acids. J. Chem. Theory Comput. (2024). 10.1021/acs.jctc.4c00732. 39151116

[50] T. J. Dolinsky , PDB2PQR: Expanding and upgrading automated preparation of biomolecular structures for molecular simulations. Nucleic Acids Res. **35**, W522–W525 (2007). 17488841 10.1093/nar/gkm276 PMC1933214

