# Supplementary material for: Structure reveals a regulation mechanism of plant outward-rectifying K+ channel GORK by structural rearrangements in the CNBD–Ankyrin bridge
Source: Proc Natl Acad Sci U S A. 2025 Jul 23;122(30):e2500070122. doi: 10.1073/pnas.2500070122 (PMC12318183; doi:10.1073/pnas.2500070122)
Supplement: Supplementary file 1 — Appendix 01 (PDF) [file pnas.2500070122.sapp.pdf]

Fig. S1

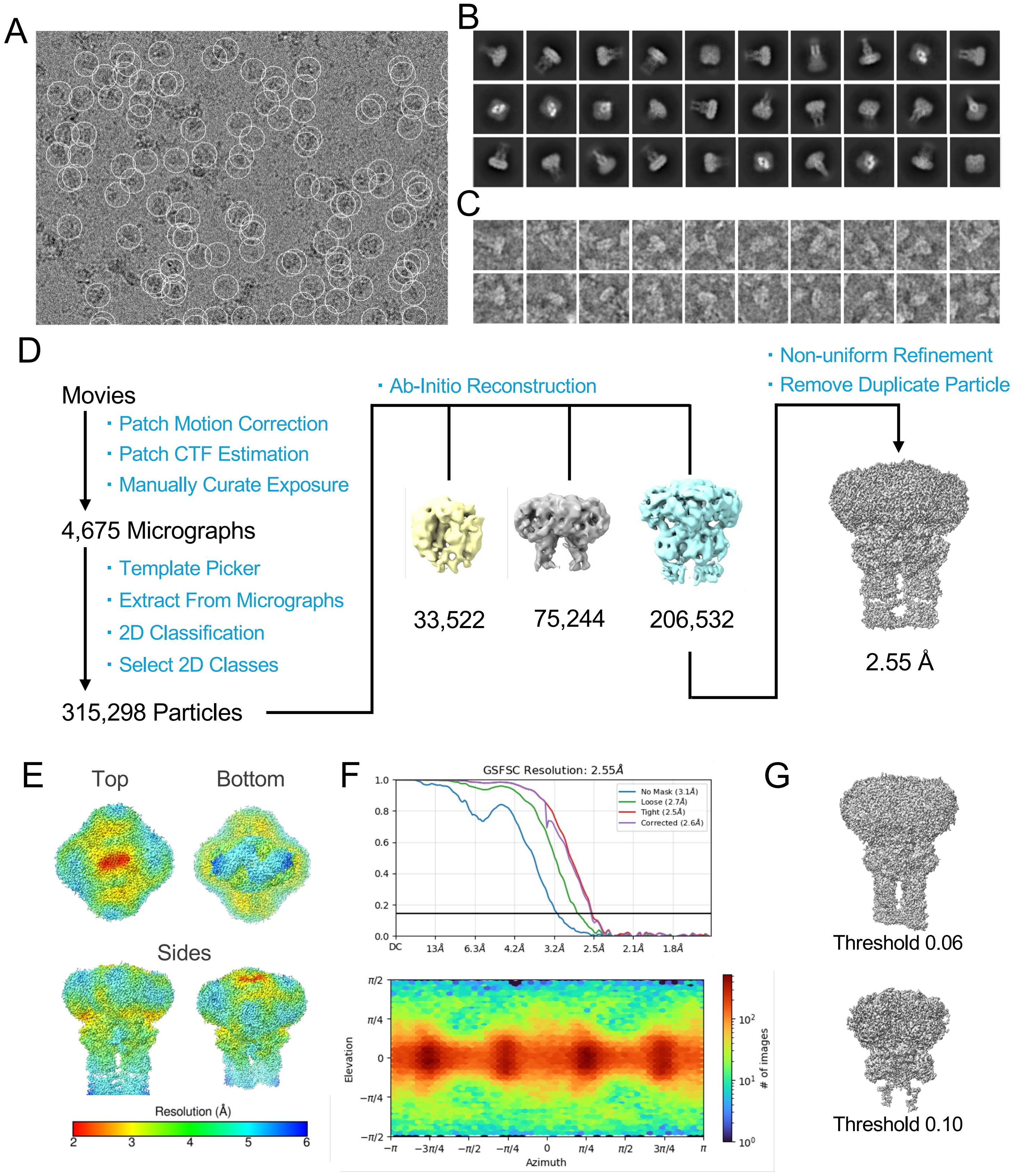

Fig. S1. Cryo-EM data processing of GORK structure
(A) Representative cryo-EM micrograph of GORK. *(B)* Representative 2D class averages of GORK. *(C)* Representative extracted particles of GORK. *(D)* Cryo-EM data processing workflow for the initial structure. *(E* and *F)* EM density colored according to resolution and Gold-standard Fourier shell correlation (FSC) curves for the final map of GORK including all particles. *(G)* Cryo-EM data of GORK.

Fig. S2

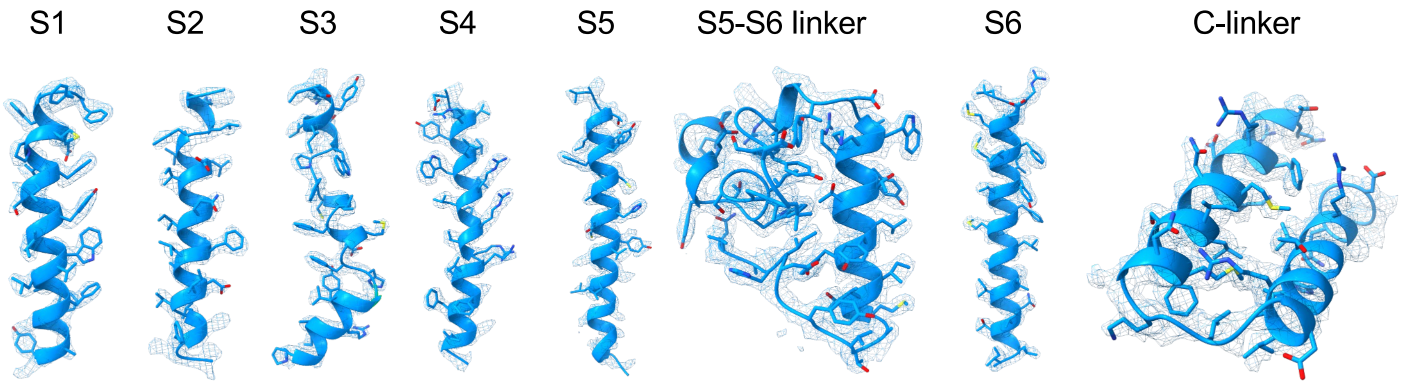

Fig. S2. The EM density maps for representative regions (S1-S6) of GORK

Fig. S3

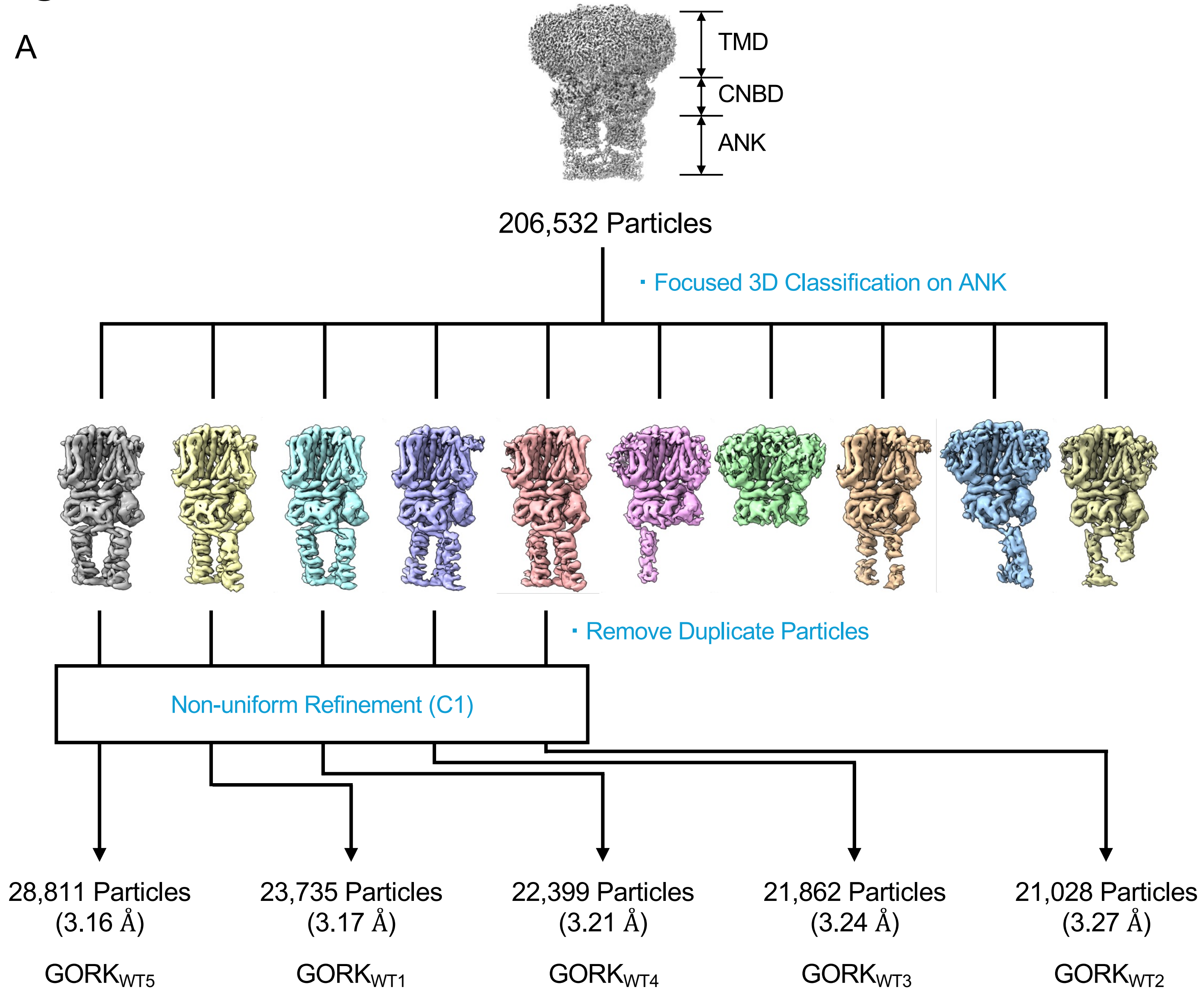

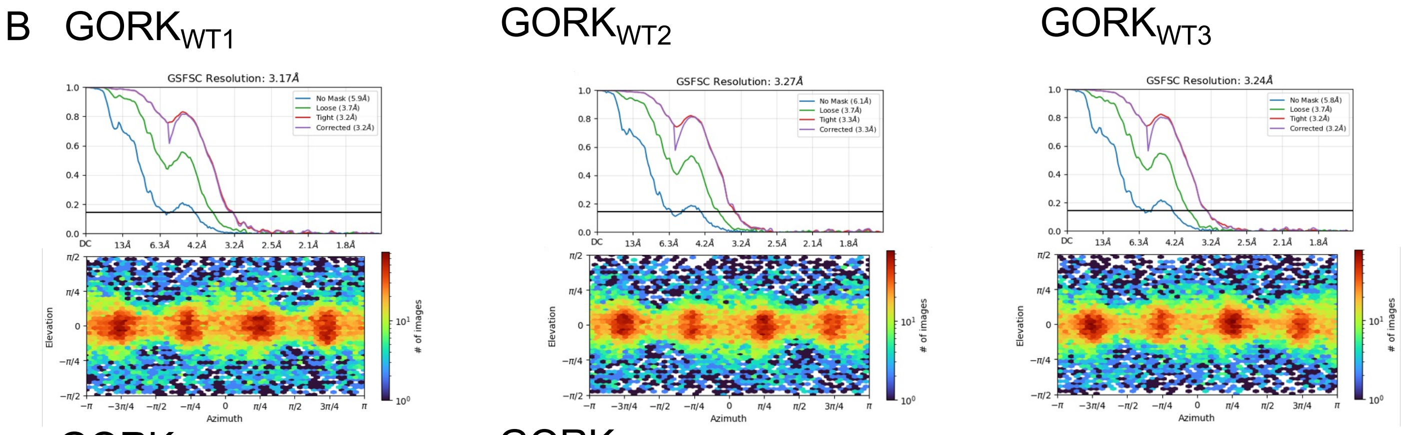

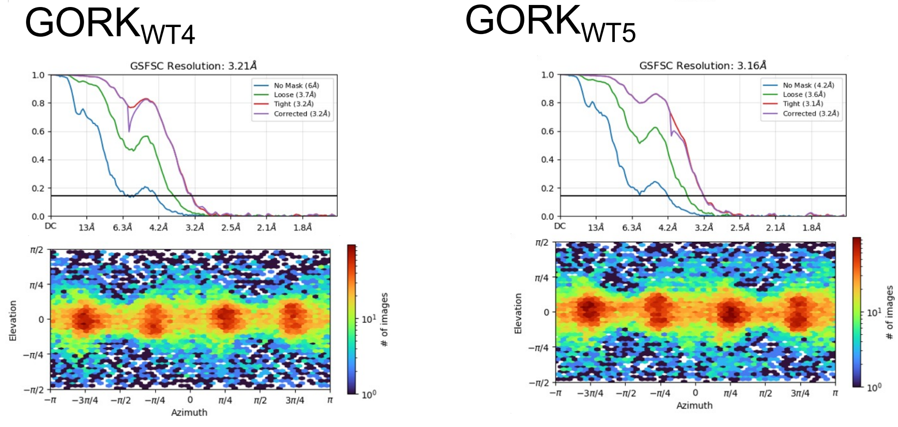

Fig. S3. Cryo-EM data processing of five different GORK structures *(A)* Focused 3D Classification workflow on particles in the first refined map. NU Refinement was performed with C1 symmetry and final maps were obtained. *(B)* FSC for each final map are indicated, with $GORK_{WT1}$, $GORK_{WT2}$, $GORK_{WT3}$, $GORK_{WT4}$ and $GORK_{WT5}$.

Fig. S4

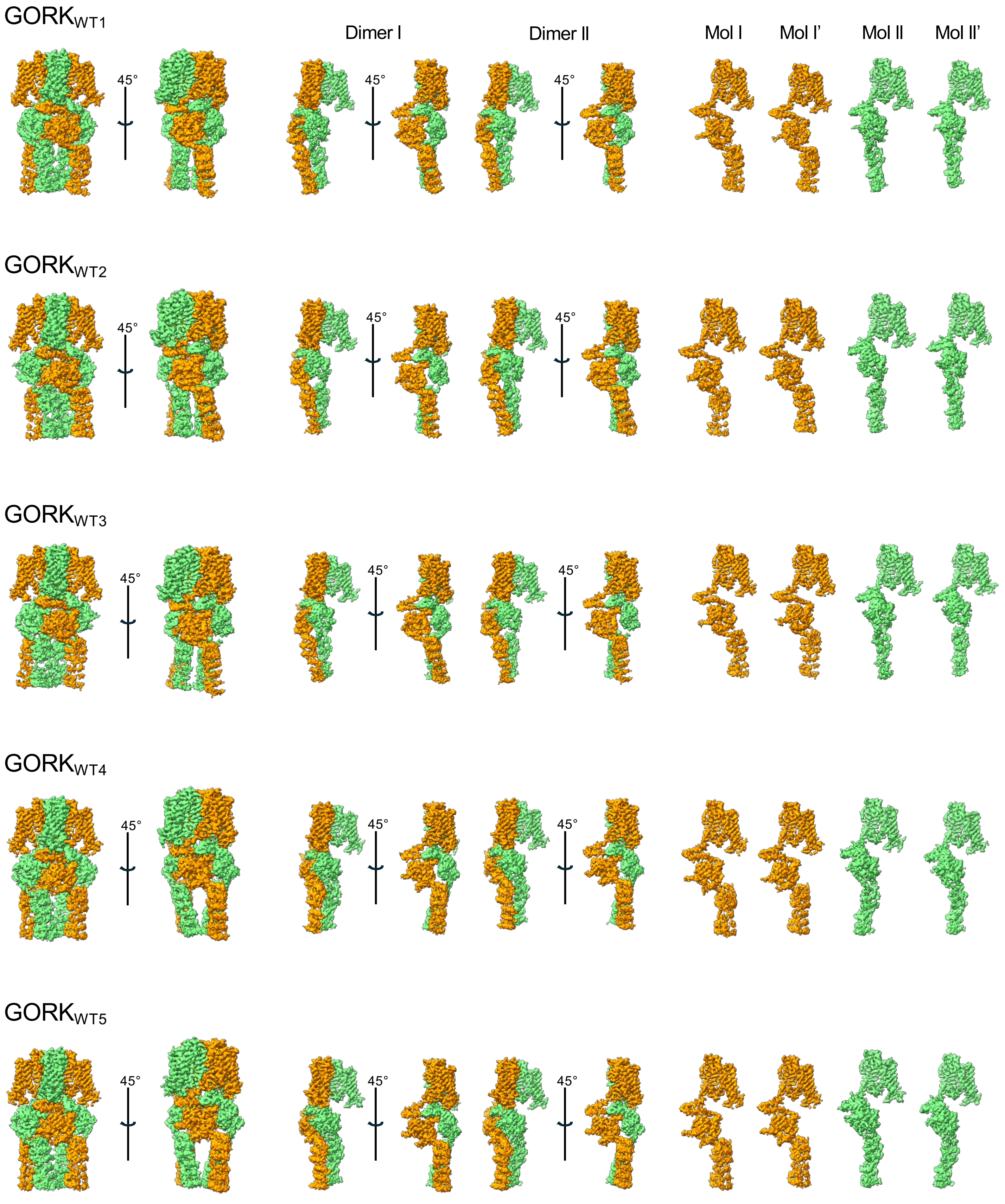

Fig. S4. Dimeric analysis of five different structures of GORK
Dimer of dimer (left), two dimers (center), and four protomers (right) for each structure, GORK$_{WT1}$, GORK$_{WT2}$, GORK$_{WT3}$, GORK$_{WT4}$ and GORK$_{WT5}$. To indicate the differences of dimers, each dimer was colored green or purple.

Fig. S5

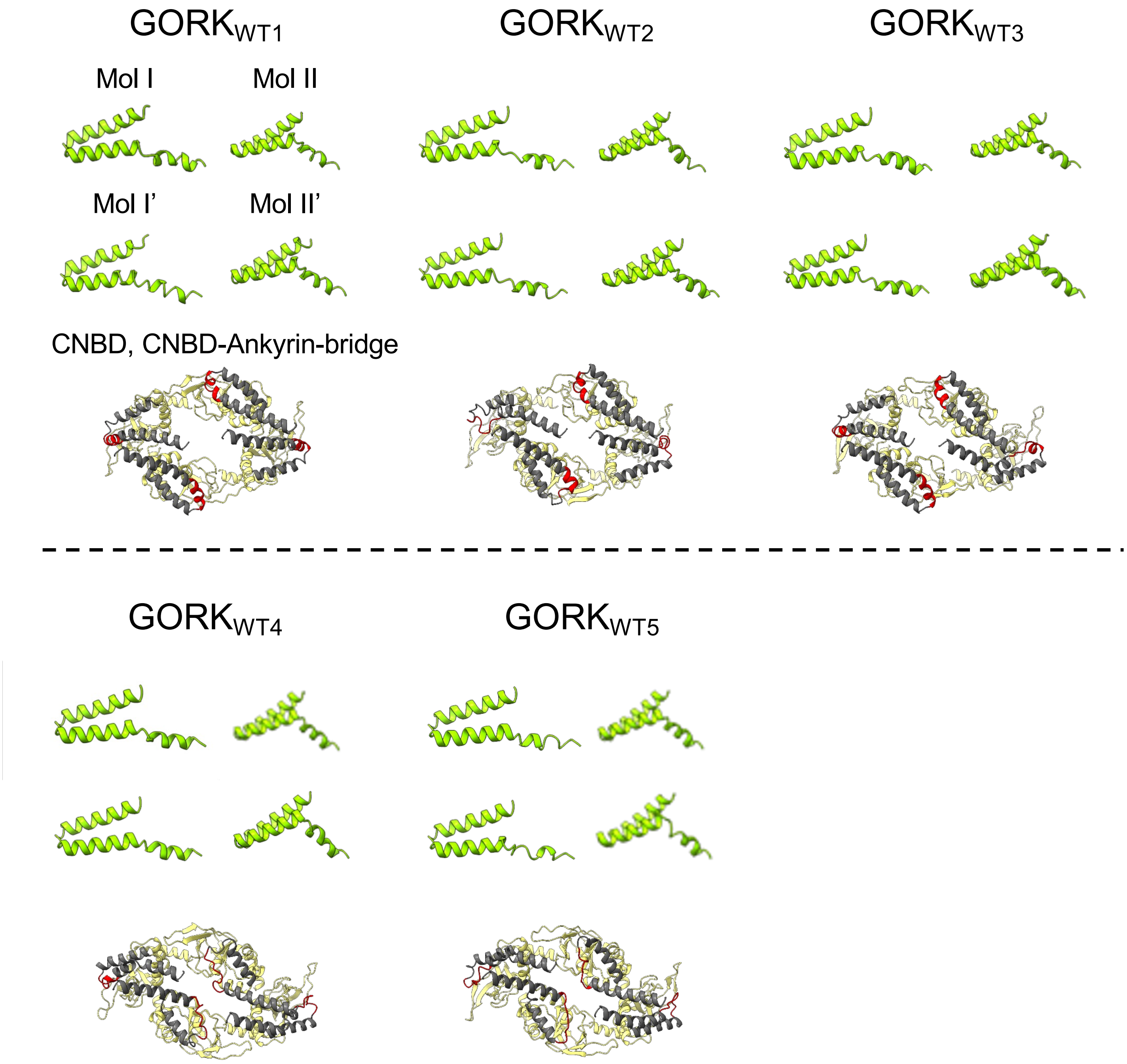

Fig. S5. Flexibility and symmetry of C-linker, CNBD and CNBD-Ankyrin-bridge of GORK
C-linkers from four protomers (top). CNBD and CNBD-Ankyrin-bridge (bottom) of GORK$_{WT1}$ (same as Fig. 3*D* and *E*), GORK$_{WT2}$, GORK$_{WT3}$, GORK$_{WT4}$ and GORK$_{WT5}$ (same as Fig. 3*D* and *E*).

Fig. S6

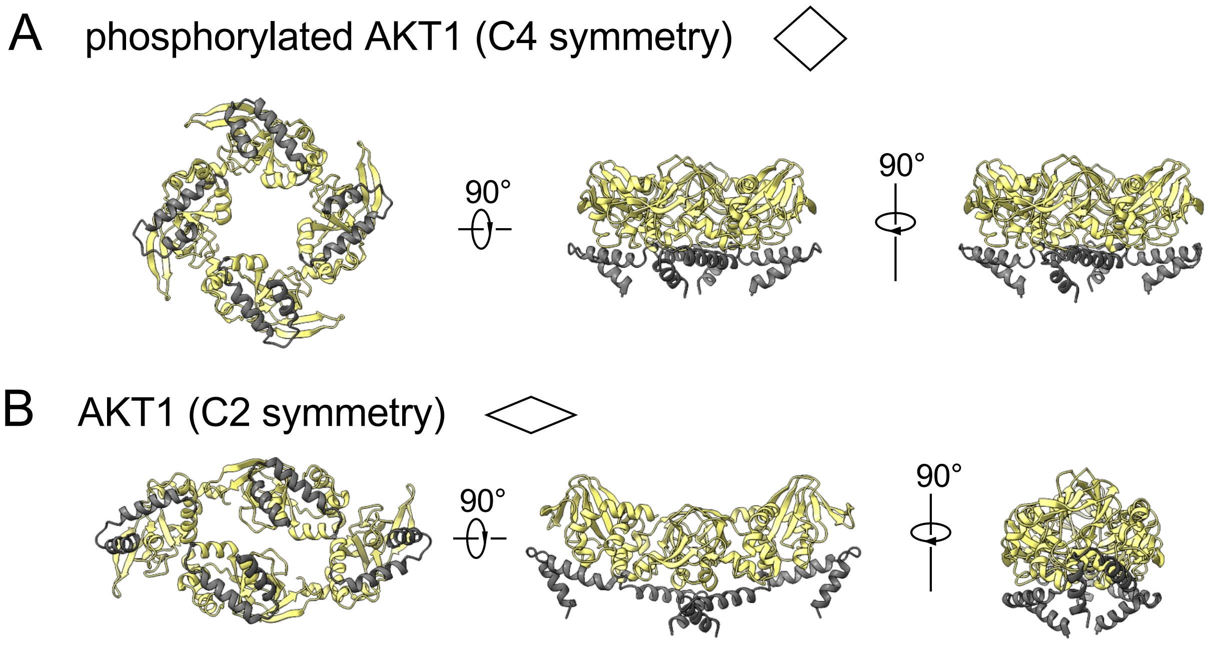

Fig. S6. CNBD-Ankyrin-bridge and CNBD in phosphorylated AKT1 (C4 symmetry) and AKT1 (C2 symmetry)

Cytosolic view (left), and side views (center and right) of the CNBD-Ankyrin-bridge and the CNBD in phosphorylated AKT1 (C4, PDB: 7FCV) *(A)* and AKT1 (C2, PDB: 7WSW) *(B)*.

Fig. S7

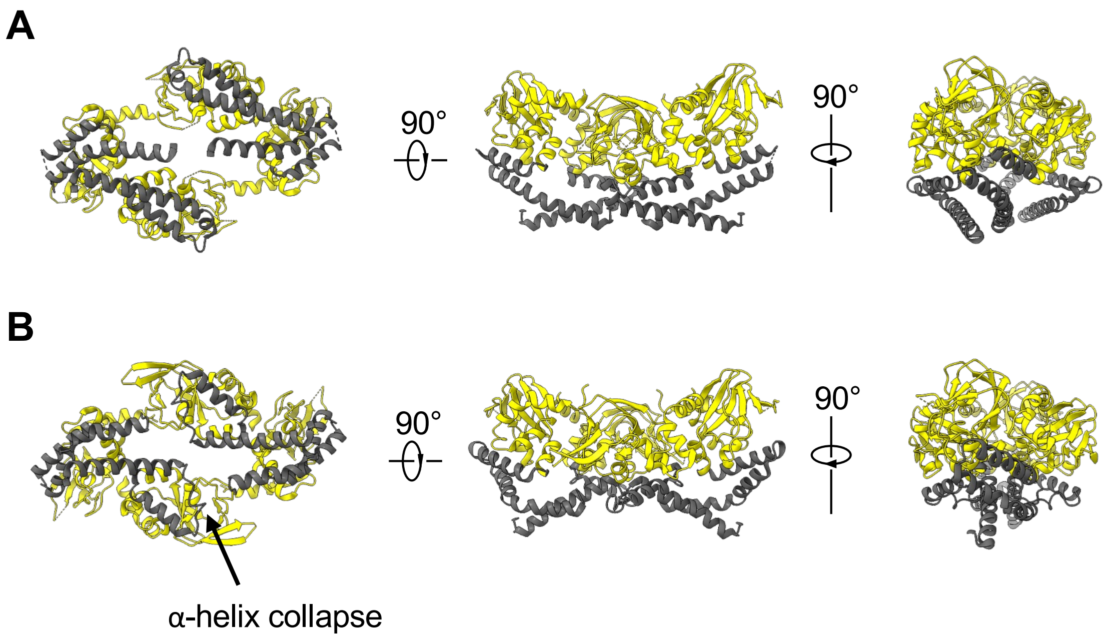

Fig. S7. CNBD-Ankyrin-bridge and CNBD in SKOR
Cytosolic view (left), and side views (center and right) of the CNBD-Ankyrin-bridge and the CNBD in SKOR similar to GORKWT1 (PDB: 8JET) *(A)* and GORKWT5 (PDB: 8JEU) *(B)*. The modeling was performed using the SKOR data (25).

Fig. S8

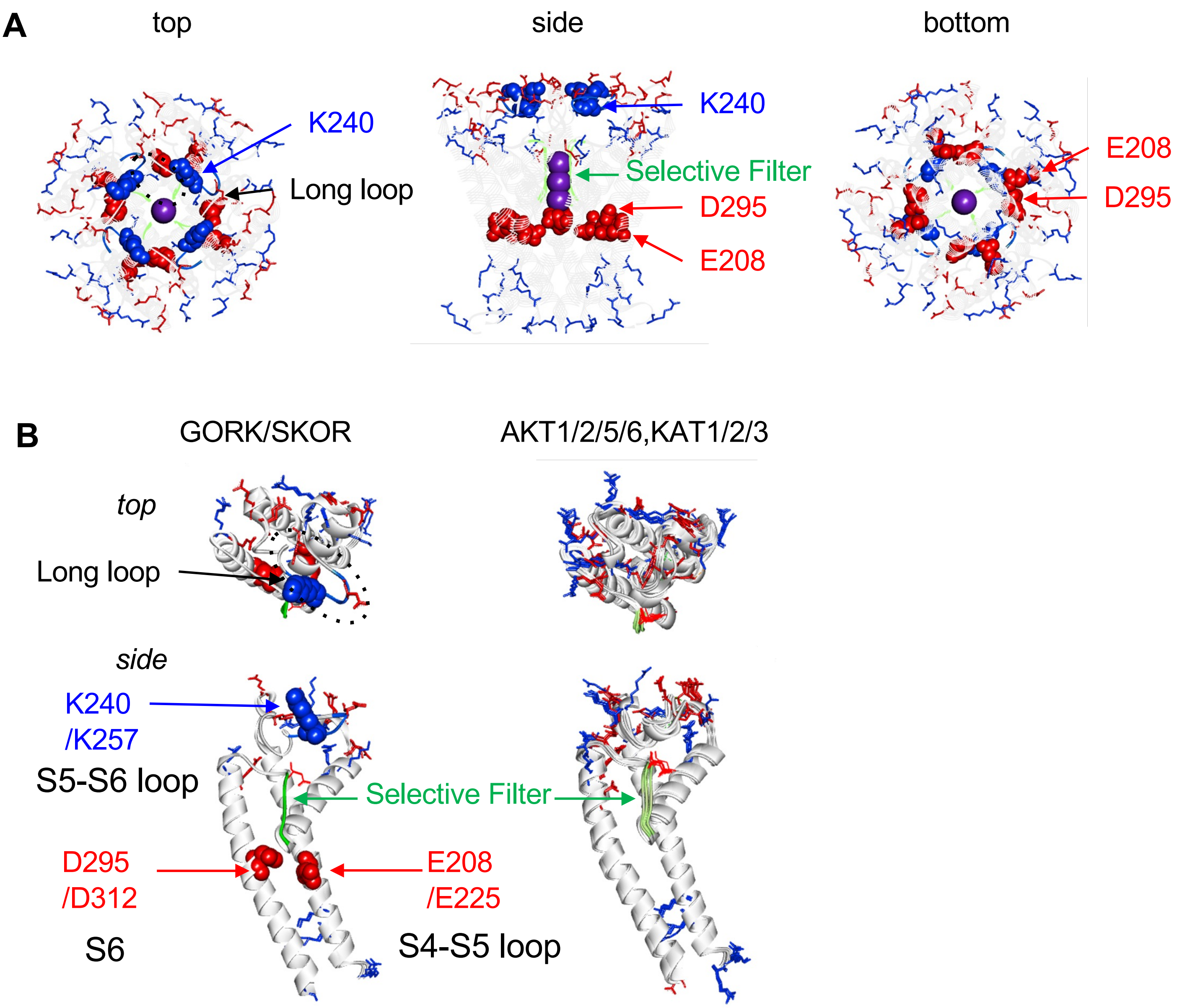

Fig. S8. Charged residues located near the pore region of GORK.
(A) Top, side, and bottom views of the pore region of GORK. K/R and D/E residues are represented in blue and red, respectively. Putative K^+ ions (purple) are placed on the selective filter (green). K240, located above the filter, and D295/E208, located below it, are represented by spheres. *(B)* Comparison of the TMD subunits of GORK/SKOR with those of AKT1/2/5/6 and KAT1/2 and AtKC1 (viewed from the pore side). The structures were obtained from the AlphaFold database (https://alphafold.ebi.ac.uk/), GORK: Q94A76, SKOR: Q9M8S6, AKT1: Q38998, AKT2: Q38898, AKT5: Q9SCX5, AKT6: Q8GXE6, KAT1: Q39128, KAT2: Q38849 and KAT3: P92960. Charged residues and filters are represented as in *(A)*.

Fig. S9

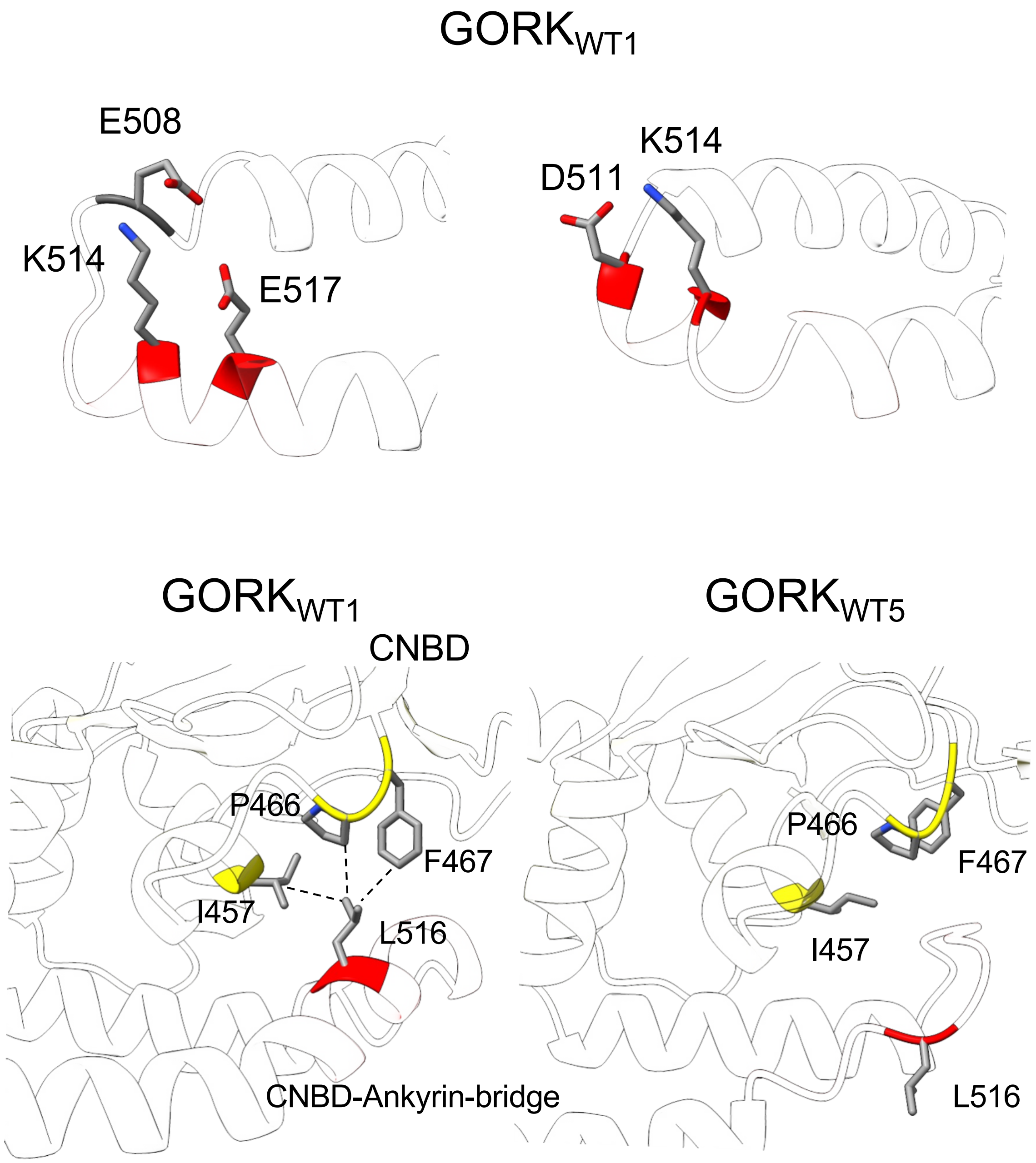

Fig. S9. The interaction of K514 and L516 with other residues.
(A) Electrostatic interactions of K514 in GORKWT1 with E508 and E517 (left) and with D511 (right). These interactions are not observed in GORK$_{WT5}$. *(B)* Hydrophobic interactions of L516 with I457, F467 and P466 in GORK$_{WT1}$ (left). In GORK$_{WT5}$ (right) the side chain of L516 points in the opposite direction with respect to those three hydrophobic residues.

Fig. S10

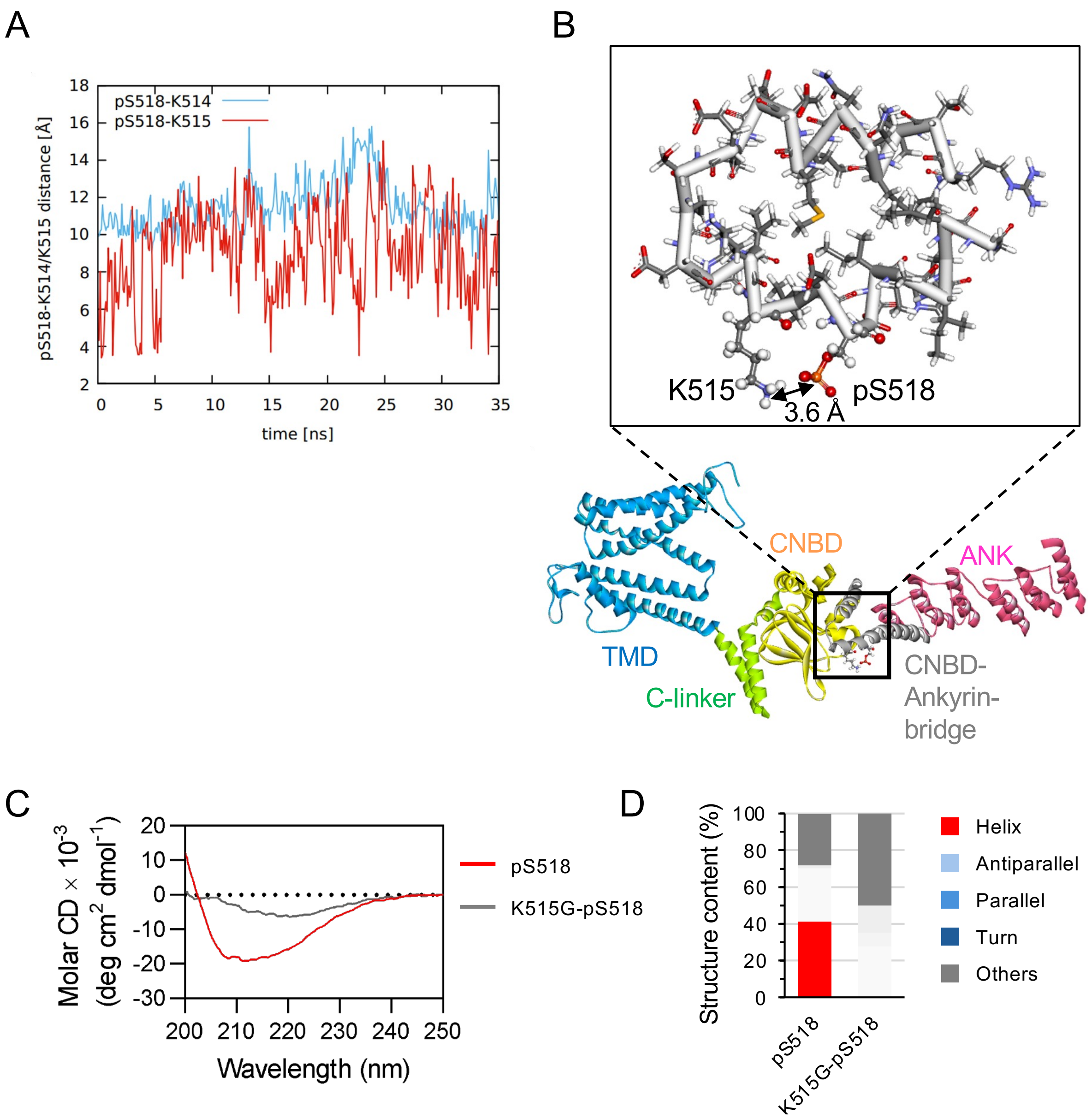

Fig. S10. Possible interaction of K515 with phosphorylated S518.
(A) Time course of the distance between pS518 and K514 or K515 in MD simulation. *(B)* Snapshot structure of the CNBD-Ankyrin-bridge at 34.1 ns in MD simulation, indicating the interaction between K515 and pS518. *(C)* CD spectra of the pS518 (red) peptide and K515G-pS518 peptide (black). Peptides were measured in 1 mM phosphate buffer containing 50 mM KCl (adjusted to pH 6.0 with KOH). *(D)* Secondary structure analysis of the CD data of panel C by BeStSel (https://bestsel.elte.hu/index.php). The elements in the bar charts are color-coded as follows: Helix (red), antiparallel (light blue), parallel (blue), turn (dark blue), and others (grey).

Table. S1

Table S1. Cryo-EM data collection, refinement, and validation statistics

**Dataset**	GORK (PDB: 9LA7)	GORK$_{WT1}$ (PDB: 9L9U)	GORK$_{WT2}$ (PDB: 9LA2)	GORK$_{WT3}$ (PDB: 9LA3)	GORK$_{WT4}$ (PDB: 9LA1)	GORK$_{WT5}$ (PDB: 9LA0)
**Data collection and processing**						
Magnification	60,000	60,000	60,000	60,000	60,000	60,000
Voltage (kV)	300	300	300	300	300	300
Electron exposure (e–/Å2)	60	60	60	60	60	60
Defocus range (μm)	-1.2 to -2.0	-1.2 to -2.0	-1.2 to -2.0	-1.2 to -2.0	-1.2 to -2.0	-1.2 to -2.0
Pixel size (Å)	0.788	0.788	0.788	0.788	0.788	0.788
Symmetry	C2	C1	C1	C1	C1	C1
Final particle images (no.)	186,257	23,735	21,028	21,862	22,399	28,811
Map resolution (Å)	2.55	3.17	3.27	3.24	3.21	3.16
FSC threshold	0.143	0.143	0.143	0.143	0.143	0.143
**Refinement**						
Map sharpening B factor (Å2)	-69.2	-38.2	-33.4	-35.3	-36.0	-41.5
Model composition						
Non-hydrogen atoms	10,340	21,836	21,917	21,944	21,940	21,944
Protein residues	1,220	2,692	2,701	2,704	2,703	2,704
Protein B factors (Å2)	60.40	168.07	183.77	156.06	134.10	147.35
R.m.s. deviations						
Bond lengths (Å)	0.005	0.005	0.006	0.005	0.006	0.006
Bond angles (°)	0.748	0.859	0.860	0.814	0.864	0.850
Validation						
MolProbity score	2.06	2.45	2.51	2.47	2.51	2.46
Clashscore	9.51	23.96	23.46	23.14	21.48	21.07
Ramachandran plot						
Favored (%)	97.36	96.31	95.88	96.51	95.51	96.48
Allowed (%)	2.64	3.69	4.12	3.49	4.49	3.52
Disallowed (%)	0	0	0	0	0	0
Rotamer outliers (%)	3.74	2.74	2.98	3.27	3.10	3.43

Movie. S1. Horizontal view of tetramer

Movie. S2. Vertical view of CNBD-Ankyrin-bridge and CNBD

Movie. S3. Horizontal view of CNBD-Ankyrin-bridge and CNBD

Movie. S4. Horizontal view of tetramer I

Movie. S5. Horizontal view of tetramer II
